# Genome-Wide Identification of Basic Helix–Loop–Helix (*bHLH*) Family in Peanut: Potential Regulatory Roles in Iron Homeostasis

**DOI:** 10.3390/ijms252212057

**Published:** 2024-11-09

**Authors:** Gangrong Shi, Zheng Zhang, Jinxiu Li

**Affiliations:** College of Life Sciences, Huaibei Normal University, Huaibei 235000, China; swx@chnu.edu.cn (Z.Z.); 12111070720@chnu.edu.cn (J.L.)

**Keywords:** *Arachis hypogaea*, genome-wide identification, *bHLH* genes, iron deficiency, cultivar difference

## Abstract

The basic helix–loop–helix (bHLH) superfamily is the second-largest transcription factor family that participates in a wide range of biological processes in plants, including iron homeostasis. Although the family has been studied in several plant species, a comprehensive investigation is still needed for peanut (*Arachis hypogaea*). Here, a genome-wide analysis identified 373 *AhbHLH* genes in peanut, which were divided into 14 groups or subfamilies according to phylogenetic analysis. Clustered members generally share similar gene/protein structures, supporting the evolutionary relationships among AhbHLH proteins. Most *AhbHLH*s experienced whole-genome or segmental duplication. The majority of AhbHLH proteins had a typical bHLH domain, while several phylogenetic groups, including Group VI, X, XIII, and XIV, had the HLH domain. The expression of several *AhbHLH* genes, including *AhbHLH001.3*, *AhbHLH029.1*/*.2*, *AhbHLH047.1*/*.2*, *AhbHLH115.1*/*.2*, *AhbHLH097.1*/*.2*, *AhbHLH109.4*, and *AhbHLH135.1*, was induced by Fe deficiency for both cultivars, or at least in Silihong, suggesting an important role in the Fe deficiency response in peanut. Nine genes (*AhbHLH001.3*, *AhbHLH029.1*/*.2*, *AhbHLH047.1*/*.2*, *AhbHLH097.1*/*.2*, and *AhbHLH115.1*/*.2*) were specifically induced by Fe deficiency in Silihong, and their expression was higher in Silihong than that in Fenghua 1. These genes might be responsible for higher tolerance to Fe deficiency in Silihong. Our findings provide comprehensive information for further elucidating the regulatory mechanism of Fe homeostasis in peanut.

## 1. Introduction

The basic helix–loop–helix (bHLH) superfamily is the second-largest transcription factor family that participates in a wide range of biological processes in plants. bHLHs are universally involved in plant developmental and metabolic processes, including photomorphogenesis; the development of carpel, anther, epidermal cells, root hair, and stomata; flowering induction; signal transduction; and secondary metabolism, and also play important roles in the plant response to diverse environmental stress, such as drought, salinity, hypothermia, and iron (Fe) deficiency [1,2].

bHLH proteins contain a highly conserved bHLH domain including a basic region and a helix–loop–helix (HLH) region, which consists of approximately 60 conserved amino acid residues [3]. The basic region usually binds to DNA motifs of the target genes such as E-box (CANNTG) and G-box (CACGTG). The HLH region, which is composed of two alpha helices separated by a variable loop, is required for the formation of homodimers and/or heterodimers [3].

Eukaryotic bHLH transcription factors are divided into six groups according to the phylogenetic relationship and DNA binding function [4,5]. Group A members specifically bind to the E-box variant (CAGCTG or CACCTG). Group B members can bind to the CACGTG or CATGTTG motif, and the G-box (CACGTG). Group C members contain a PAS domain that can bind to non-E-box (NACGTG or NGCGTG) core sequences. Group D members lack a basic region and are mainly involved in heterodimerization with other bHLH proteins rather than binding to DNA. Group E members can preferentially bind to N-boxes (CACGGC or CACGAC). Group F members contain more than one COE domain that is involved in COE (Col/Olf-1/EBF) dimerization and DNA binding. In plants, Group B members are known to be the main bHLH proteins, while Group F members have not been identified. At least 162 bHLH proteins have been identified in *Arabidopsis* and are divided into 12 subfamilies [3,6]. Although most of the bHLHs in *Arabidopsis* show close phylogenetic relationships with Group B members of eukaryotic bHLHs [3,7], some atypical bHLH proteins that lack the basic region but do not share a close sequence similarity with Group D proteins have also been identified [3,8].

Iron is an essential micronutrient for the growth and development of virtually all organisms. In plants, it acts as a catalytic and structural cofactor for a large number of enzymes and plays vital roles in many metabolic processes, including respiration, photosynthesis, sulfur assimilation, and nitrogen fixation [9]. Although Fe is one of the most abundant elements in soils, its bioavailability is limited because it mainly exists in the form of highly stable insoluble oxyhydroxides [10]. The shortage of Fe inhibits plant growth and reduces crop yields. More importantly, as plant-based diets are the primary Fe source for most human populations, Fe deficiency in the edible tissues of plants is responsible for most anemia across the world. Thus, improving Fe uptake by crops and enhancing Fe content in edible parts is particularly important for both crop production and human health. It requires the physiological and molecular mechanisms of Fe uptake and translocation to be fully understood.

To cope with Fe deficiency and maintain Fe homeostasis, plants have developed a series of sophisticated systems to regulate Fe uptake and transport, in which some bHLH transcription factors play important roles [2]. In *Arabidopsis*, four bHLH IVc transcription factors (bHLH34, bHLH104, bHLH105/ILR3, and bHLH115) form homo- and heterodimers themselves [11,12,13] or interact with bHLH121/URI (upstream regulator of IRT1) [14,15,16,17] to activate the expression of bHLH Ib genes (*bHLH38*, *bHLH39*, *bHLH100*, and *bHLH101*) as well as *bHLH29* (FER-like iron-deficiency-induced transcription factor, *FIT*) and *bHLH47* (POPEYE, *PYE*). FIT interacts with bHLH Ib to form heterodimers that directly facilitate the activation of Fe-deficiency-responsive genes such as *FRO2* and *IRT1* [18,19,20]. PYE interacts with bHLH104, bHLH105, and bHLH115 to form heterodimers, which can bind to the promoters of several Fe-responsive genes such as *NAS4* (nicotianamine synthase 4) to repress their expression, consequently altering Fe translocation [11,12,21].

Peanut (*Arachis hypogaea* L., 2n = 4x = 40) is the fourth largest oilseed crop in the world, providing 20% of edible oil and 11% of food protein to the global population every year. Unfortunately, peanut productivity is always affected by Fe deficiency in soil because it is sensitive to Fe deficiency and mainly grows in calcareous soils [22]. To improve Fe nutrition in peanut, the mechanism of Fe uptake and translocation should be fully understood. For this purpose, several Fe-transport-related genes such as *AhFRO1* [23], *AhIRT1* [24], and *AhNramp1* [25] have been functionally characterized. Comparative transcriptome analysis revealed that *AhFRO2*, *AhIRT1*, *AhNramp3*, *AhYSL3*, and *AhOPT3* are involved in Fe uptake and transport in peanut under Fe deficiency conditions [26]. However, little is known about the molecular regulatory mechanisms of Fe homeostasis in peanut.

A previous study revealed that there were 132 and 129 bHLH proteins in the two wild ancestral species of the cultivated peanut, *A. duranensis* (AA) and *A. ipaënsis* (BB), respectively [27]. However, the *AhbHLH* gene family in peanut has not yet been comprehensively investigated. To fill this gap, the whole-genome identification of peanut *AhbHLH* was performed in this study and their conserved domains and motifs, structures, evolutionary relationships, and gene structures were characterized. Furthermore, the expression of some *AhbHLH* genes in response to Fe deficiency was investigated. Our data will provide a basis to further functionally characterize *AhbHLH* genes and shed new light on the possible roles of some *AhbHLH* genes in the regulation of Fe homeostasis in peanut plants.

## 2. Results

### 2.1. Identification and Phylogenetic Analysis of the AhbHLH Family in Peanut

A total of 373 putative *AhbHLH* genes were identified in peanut (Table S1). The length of the *AhbHLH* genes varied from 549 bp (*AhbHLH162.3*) to 17882 bp (*AhbHLH059.2*), with CDS lengths from 273 bp (*AhbHLH135.1*/*.4*) to 3501 bp (*AhbHLH157.2*). The amino acid number of AhbHLH proteins ranged from 90 aa (AhbHLH135.1/.4) to 1166 aa (AhbHLH157.2), and the molecular weight varied from 9.97 kDa (AhbHLH135.5) to 133.36 kDa (AhbHLH157.2). The instability index for 97% AhbHLH proteins were larger than 40, indicating low stability in vitro. The GRAVY (grand average of hydropathicity) of all AhbHLH proteins was less than 0, except AhbHLH045.1 (0.022), suggesting AhbHLHs are hydrophilic proteins. The aliphatic index and isoelectric point (pI) varied widely, ranging from 45.79 (AhbHLH123.2) to 106.96 (AhbHLH031.5), and from 4.45 (AhbHLH020.14) to 11.82 (AhbHLH147.2/.4), respectively (Table S1).

**Figure 1:**
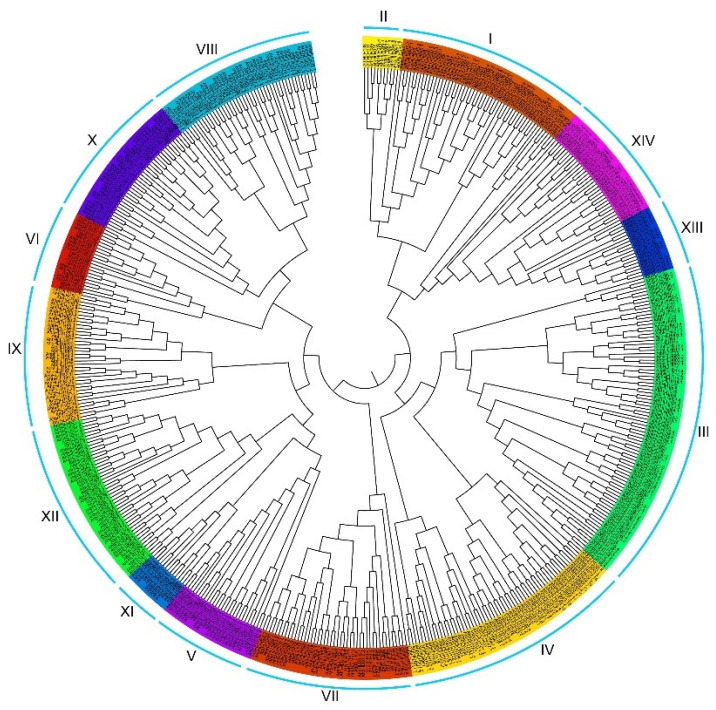
Phylogenetic relationships of bHLH proteins in peanut (AhbHLH) and *Arabidopsis thaliana* (AtbHLH). Members of the 14 *bHLH* gene groups are detailed in Table S2.

**Figure 2:**
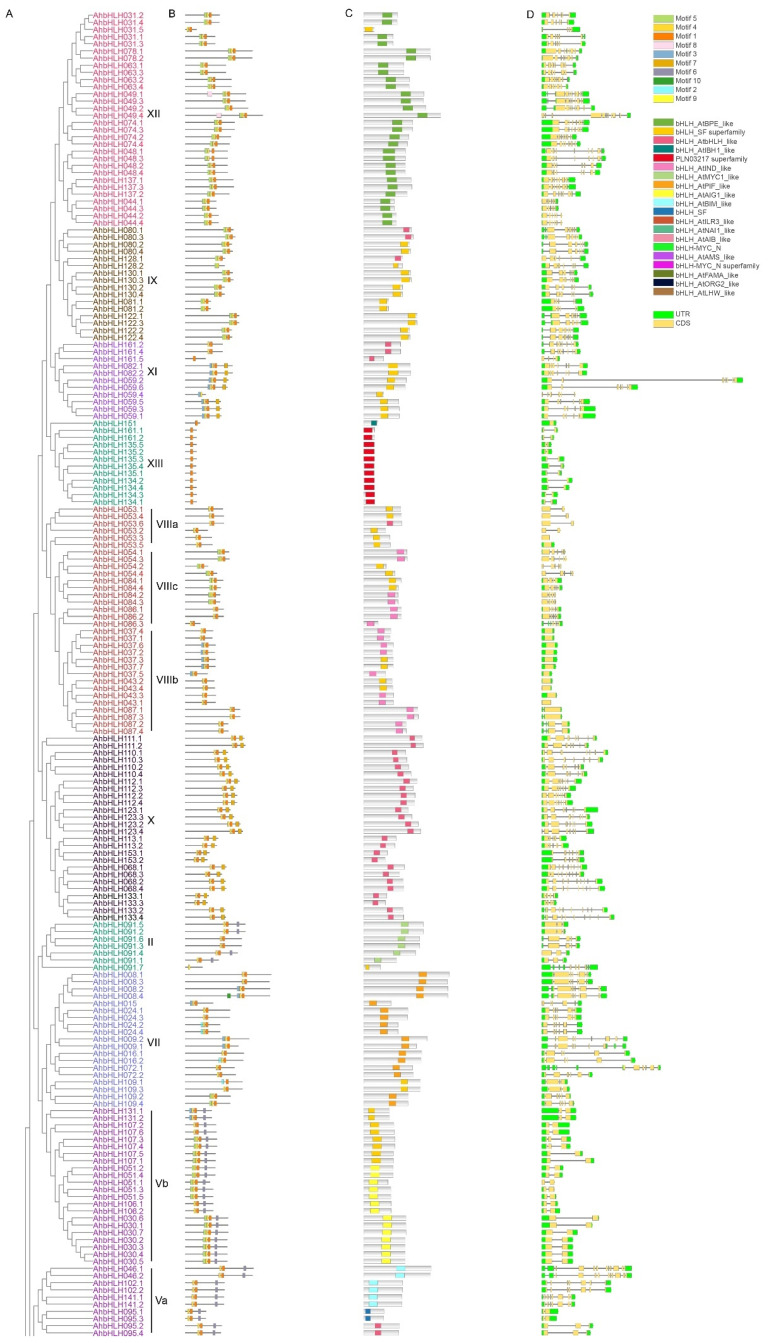

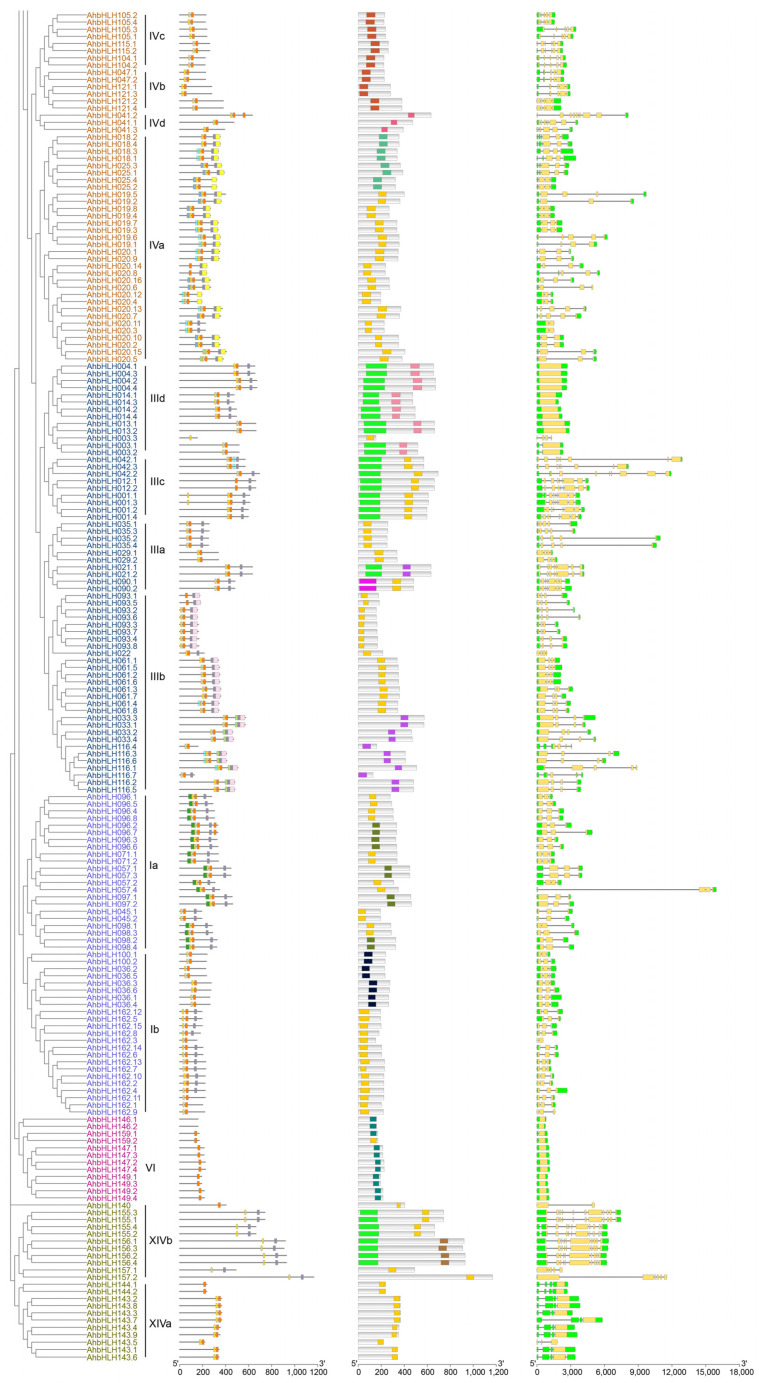
Phylogenetic relationships (**A**), conserved motifs (**B**), and domains (**C**) of AhbHLH proteins as well as gene structure (**D**) in peanut. UTR and CDS represent untranslated regions and coding sequences, respectively.

To reveal the phylogenetic relationship among *AhbHLH* genes, a phylogenetic tree was constructed with 535 bHLH protein sequences from peanut and *Arabidopsis* (Figure 1 and Figure 2A). As presented in Figure 1, *bHLH* gene members were divided into 14 groups (subfamilies), and each group contained 7 to 60 genes in peanut (Table S2). Interestingly, although the number of protein sequences in our study is three times higher than that of Heim et al. [28], the classification is identical except Group VI and VII, which are merged into Group VII in this study. In addition, three novel groups (Group VI, XIII, and XIV) were identified (Figure 1, Table S2). Group VI contained eight *Arabidopsis* AtbHLH proteins (AtbHLH146/147/148/149/150/151/158/159) and 13 peanut AhbHLH proteins (AhbHLH146.1/.2, AhbHLH147.1/.2/.3/.4, AhbHLH149.1/.2/.3/.4, AhbHLH151, AhbHLH159.1/.2). Group XIII consisted of four *Arabidopsis* AtbHLH proteins (AtbHLH134/135/136/161) and 14 peanut AhbHLH proteins (AhbHLH134.1/.2/.3/.4, AhbHLH135.1/.2/.3/.4/.5, AhbHLH161.1/.2/.3/.4/.5). Group XIV consisted of nine *Arabidopsis* AtbHLH proteins (AtbHLH142/143/144/145/152/155/156/157/160) and 22 peanut AhbHLH proteins (AhbHLH140, AhbHLH143.1/.2/.3/.4/.5/.6/.7/.8/.9, AhbHLH144.1/.2, AhbHLH155.1/.2/.3/.4, AhbHLH156.1/.2/.3/.4, AhbHLH157.1/.2).

### 2.2. Conserved Motifs, Domain Architectures, and Gene Structure

Ten conserved motifs were identified in AhbHLH proteins (Figure 2B and Table S3). All AhbHLH proteins shared motifs 1 and/or 4 except AhbHLH146.1/.2 (without motif). Motif 5 was widely distributed in several groups including Groups IX, XI, and XII, while Motif 6 was frequently observed in Groups I, II, III, IV, V, and XIV. Motifs 7, 8, 9, and 10 were specifically distributed in Groups X (and XI), III, IV, and I, respectively. The distribution pattern of conserved motifs varied among phylogenetic groups, whereas it was generally similar within the same group (Figure 2B).

All AhbHLH proteins contained the typical domain named bHLH (Figure 2C). According to the CDD tool, the conserved domains were divided into 17 group-specific functional types: bHLH_AtBPE_like (Group XII), bHLH_AtbHLH_like (Group IX, X, and XI), bHLH_AtIBH1_like (Group VI), PLN03217 superfamily (Group XIII), bHLH_AtIND_like (Group VIII), bHLH_AtMYC1_like (Group II), bHLH_AtPIF_like (Group VII), bHLH_AtAIG1_like (Group V), bHLH_AtBIM_like (Group V), bHLH_AtILR3_like (Group IV), bHLH_AtNAI1_like (Group IV), bHLH_AtAIB_like (Group III), bHLH-MYC_N (Group III), bHLH_AtAMS_like (Group III), bHLH_AtFAMA_like (Group I), bHLH_AtORG2_like (Group I), and bHLH_AtLHW_like (Group XIV).

Significant variation was observed in the exon–intron organization among *AhbHLH* genes (Figure 2D). The number of exons and introns ranged from 1 to 11 and from 0 to 10, respectively. The exon–intron organization was generally similar within most of the 14 phylogenetic groups. Although large variations of exon–intron organization were detected in Group III, IV, V, VII, and VIII, it was similar in the subgroups. Therefore, the exon–intron organization further supports the phylogenetic groups or subgroups defined here.

### 2.3. Multiple Sequence Alignment of AhbHLH Proteins

The multiple sequence alignment revealed that, although the majority of AhbHLH proteins had a typical bHLH domain, it was distinctive in several phylogenetic groups including Group VI, X, XIII, and XIV (Figure S1). The diversity of the bHLH domains was consistent with the results of the conservative motif and domain analysis.

**Figure 3:**
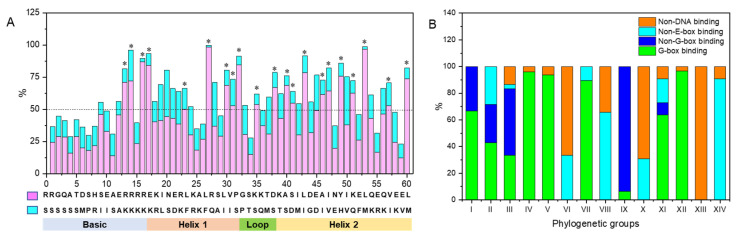
The frequencies for the most common amino acid in each position across the bHLH domain (**A**) and proportions of the predicted DNA-binding characteristics (**B**) of AhbHLH proteins based on multiple sequence alignments shown in Supplementary Figure S1. Asterisks above the bars indicate that the conservation of the most frequent amino acid is larger than 50% across all members.

The bHLH domain sequences of AhbHLH proteins contained 21 residues with at least 50% conservation across all members (Figure 3A). The basic region had four conserved residues (Glu-13, Arg-14, Arg-16, and Arg-17). The first helix region had five conserved residues (Arg-23, Leu-27, Leu-30, Val-31, and Pro-32), while the second helix region had ten conserved residues (Ala-40, Ser-41, Leu-43, Ala-46, Ile-47, Tyr-49, Lys-51, Leu-53, Val-57, and Leu-60). In contrast, only two conserved residues (Lys-36 and Asp-38) were detected in the loop region. Among these conserved residues, ten were present in more than 70% of sequences, including four Leu (Leu-27, 43, 53, and 60) and three Arg (Arg-14, 16, and 17), as well as Glu-13, Pro-32, and Tyr-49, suggesting crucial roles for the function of bHLH proteins.

The DNA-binding activity of bHLH proteins is determined by the basic region of bHLH domains [3]. Based on the presence of Glu-13 and Arg-16 in the basic region, 247 E-box-binding AhbHLH proteins (66.2%) were identified in the peanut genome (Figure 3B and Table S4). Among them, 184 G-box binders were identified according to the presence of His/Lys-9, Glu-13, and Arg-17 residues in the basic region. The remaining AhbHLH proteins were further divided into two categories, i.e., 61 non-E-box DNA-binding proteins (five to nine basic residues) and 65 non-DNA-binding proteins (basic residues less than five).

As for the phylogenetic groups, Groups I, II, III, IV, V, VII, IX, XI, and XII are mainly composed of E-box-binding proteins, while no E-box-binding proteins were found in Groups VI, VIII, XIII, and XIV (Figure 3B). The G-box binders form the majority of Groups IV, V, VII, and XII and part of Groups I, II, III, IX, and XI. The non-G-box binders form the majority of Group IX and part of Groups I, II, III, and XI. The non-DNA-binding proteins were found in all phylogenetic groups except Groups I, II, and IX. These data indicate that the different phylogenetic groups may have evolved different physiological functions based on their specific recognition of DNA binding sites.

### 2.4. Chromosome Location, Gene Duplication, and Ka/Ks of the AhbHLH Family

**Figure 4:**
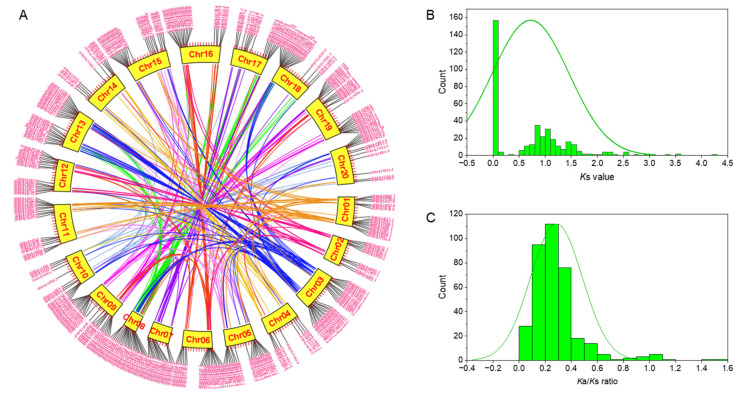
Collinear blocks (**A**) and distribution of *Ks* (**B**) and *Ka*/*Ks* (**C**) of duplicated peanut *AhbHLH* genes obtained from collinearity analysis.

The 373 *AhbHLH* genes were located unevenly in 20 chromosomes, and subgenomes A (Chr. 01–10) and B (Chr. 11–20) possess 184 and 189 *AhbHLH* genes, respectively (Figure 4A). Chromosome 03 contained the largest number of *AhbHLH* genes (28), followed by chromosome 09 (26) and 13 (26), while chromosome 09 and 14 only contained 9 and 10 *AhbHLH* genes, respectively. Most of the *AhbHLH* genes experienced gene duplication events, resulting in 389 collinear blocks (Figure 4A). Among them, 271 collinear blocks occurred between the two subgenomes, which could be considered whole-genome duplications (WGDs), while 108 collinear blocks occurred within subgenomes A or B, which could be considered segmental duplication. Additionally, ten collinear blocks of *AhbHLH* genes were identified as tandem duplications.

To detect potential events of WGD or segmental duplicated *AhbHLH* genes, values of *Ka* (the number of nonsynonymous substitutions per nonsynonymous site) and *Ks* (the number of synonymous substitutions per synonymous site) within paralogous pairs were determined (Table S5). The *Ks* value ranged from 0.00 to 3.51 with an average of 0.54 for WGD, and from 0.57 to 4.26 with an average of 1.25 for segmental duplications, respectively. The distribution of *Ks* revealed two significant *Ks* peaks in paralogous pairs (Figure 4B). One is near 0.0 and accounts for 41.64% of the paralogous pairs, which might represent the recent WGD from the allopolyploidization event. The other smaller peaks near 1.0 might represent the latest ancient WGD event, from which the majority of the segmental duplication occurred (Figure 4B).

The majority of the duplicated gene pairs had *Ka*/*Ks* ratios significantly less than 1 (Figure 4C and Table S5), indicating that the *AhbHLH* gene family is principally subject to purifying selection [29]. However, five gene pairs (*AhbHLH123.1*/*123.3*, *AhbHLH133.1*/*133.3*, *AhbHLH053.1*/*053.6*, *AhbHLH053.3*/*053.7*, *AhbHLH135.1*/*135.3*) with *Ka*/*Ks* ratios of 1 are under neutral selection, and nine gene pairs (*AhbHLH044.1*/*044.3*, *AhbHLH093.4*/*093.8*, *AhbHLH143.1*/*143.6*, *AhbHLH080.1*/*080.3*, *AhbHLH093.3*/*093.7*, *AhbHLH146.1*/*146.2*, *AhbHLH086.1*/*086.2*, *AhbHLH149.1*/*149.3*, *AhbHLH130.1*/*130.3*) with *Ka*/*Ks* ratios higher than 1 are subjected to positive selection.

### 2.5. Differentially Expressed AhbHLH Genes Under Fe Deficiency

**Figure 5:**
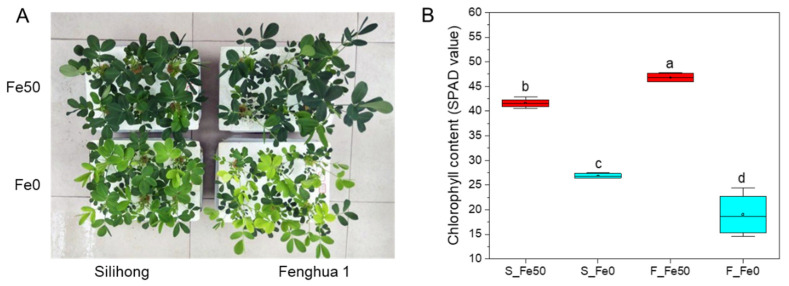
Plant morphology (**A**) and leaf chlorophyll contents (SPAD values) (**B**) of Silihong (S) and Fenghua 1 (F) grown under Fe-sufficient (Fe50) and -deficient (Fe0) conditions for 14 days. Data (means ± SE, *n* = 4) sharing the same letter(s) above the error bars are not significantly different at the 0.05 level based on the Duncan multiple range test.

To understand the responses of *AhbHLH* genes to Fe deficiency, two peanut cultivars, Silihong (Fe-deficiency-tolerant cultivar, S) and Fenghua 1 (Fe-deficiency-sensitive cultivar, F), were used for RNA-seq based comparative transcriptome analysis (Figure 5). As shown in Figure 5, Fe deficiency significantly reduced the plant growth and leaf chlorophyll contents (SPAD values) for both cultivars. By contrast, the reduction in SPAD values were considerably higher in Fenghua 1 (59%) than in Silihong (23%), indicating that Silihong is more tolerant to Fe deficiency than Fenghua 1.

**Figure 6:**
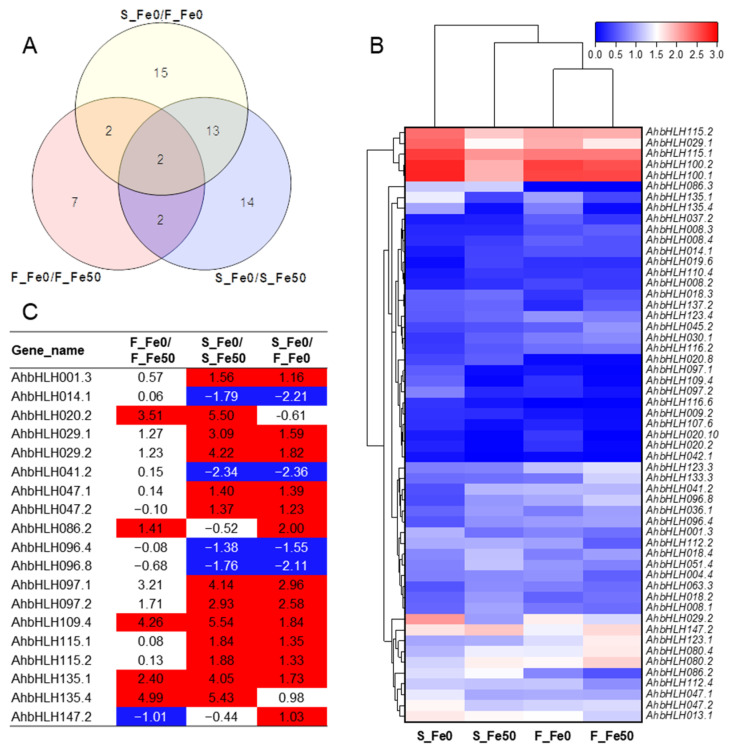
Identification of DGEs from the *AhbHLH* gene family in Fenghua 1 (F) and Silihong (S) under Fe-sufficient (Fe50) and Fe-deficient (Fe0) conditions. (**A**) Venn diagrams of the 55 DEGs among three comparisons. (**B**) Heatmap and hierarchical clustering analysis of the 55 DEGs identified in the *AhbHLH* gene family. (**C**) The log2FC values of the 19 DEGs shared by at least two comparisons. Red and blue color represents up- and downregulated DEGs, respectively.

A total of 55 differentially expressed genes (DEGs) were identified in the *AhbHLH* family; among them, 13, 31, and 32 DEGs were detected in F_Fe0/F_Fe50, S_Fe0/S_Fe50, and F_Fe0/S_Fe0, respectively (Figure 6A and Table S6). The data showed a different response of *AhbHLH* genes to Fe deficiency between the two cultivars. A total of 19 DEGs were shared by at least two comparisons, and two DEGs were shared by all comparisons (Figure 6A).

The heat map analysis revealed that the 55 DEGs could be divided into three distinct clusters, representing high, medium, and low expression levels respectively (Figure 5B). Cluster I contained five highly expressed genes including *AhbHLH029.1*, *AhbHLH100.1*/*.2*, and *AhbHLH115.1*/*.2* (Figure 5B). Cluster III contained ten genes with medium expression levels including *AhbHLH013.1*, *AhbHLH029.2*, *AhbHLH047.1*/*.2*, *AhbHLH080.2*/*.4*, *AhbHLH086.2*, *AhbHLH112.4*, *AhbHLH123.1*, and *AhbHLH147.2* (Figure 6B). Cluster II included the remaining 40 DEGs with low expression (Figure 6B).

Among the 19 DEGs that were shared by at least two comparisons, four genes (*AhbHLH020.2*, *AhbHLH109.4*, and *AhbHLH135.1*/*.4*) were commonly induced by Fe deficiency for both cultivars (Figure 6C). Nine genes (*AhbHLH001.3*, *AhbHLH029.1*/*.2*, *AhbHLH047.1*/*.2*, *AhbHLH097.1*/*.2*, and *AhbHLH115.1*/*.2*) were specifically induced by Fe deficiency in Silihong, and their expression in Silihong was higher than that in Fenghua 1 (Figure 6C). In contrast, four genes (*AhbHLH014.1*, *AhbHLH041.2*, and *AhbHLH096.4*/*.8*) were specifically repressed by Fe deficiency in Silihong, showing lower expression compared with Fenghua 1. Additionally, *AhbHLH086.2* was specifically induced by Fe deficiency in Fenghua 1, while *AhbHLH147.2* was repressed (Figure 6C).

### 2.6. Co-Expression Networks in Response to Fe Deficiency

**Figure 7:**
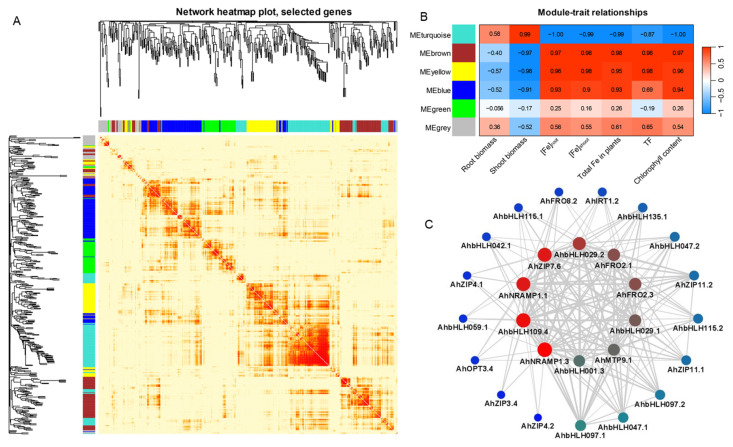
Identification of co-expression network modules via WGCNA analysis in two peanut cultivars under Fe-deficiency. (**A**) Cluster dendrogram and network heatmap of genes subjected to the co-expression module calculation. (**B**) Module-trait associations based on Pearson correlations in different cultivars or Fe treatments. The color code from blue to red represents r^2^ values ranging from −1 to 1. The TF represents translocation of Fe from roots to shoots. (**C**) Gene co-expression network of the blue module. The color code from blue to red or the size of node circle is positively correlated with the number of the interacting genes. Line width represents weight values that ranged from 0.40 to 0.53.

To identify the *AhbHLH* genes responsible for Fe deficiency resistance in peanut and reveal the possible regulatory network, a weighted correlation network analysis (WGCNA) was performed on TPM (Transcripts Per kilobase of exon model per Million mapped reads) data of 500 genes from *AhbHLH*, *AhZIP*, *AhOPT*, *AhMTP*, *AhNRAMP*, *AhHMA*, *AhNAS*, and *AhFRO* families. Six different modules were identified based on the co-expression patterns, which are marked with branches in different colors (Figure 7A). Among them, the blue module exhibited the most significant correlation with chlorophyll contents (r = 0.99) (Figure 7B). Since chlorophyll content is considered to be an important parameter indicating Fe deficiency in plants, we believe that the blue module is a Fe-deficiency-responsive module. Therefore, the blue module was further analyzed for visualization through Cytoscape 3.9.1 with a weight threshold of 0.3. As presented in Figure 7C, 15 *AhbHLH* genes were included in the constructed gene network, including *AhbHLH001.3*, *AhbHLH029.1*/*.2*, *AhbHLH036.1*, *AhbHLH042.1*, *AhbHLH047.1*/*.2*, *AhbHLH059.1*, *AhbHLH068.3*, *AhbHLH097.1*/*.2*, *AhbHLH109.4*, *AhbHLH115.1*/*.2*, and *AhbHLH135.1*. These *AhbHLH* genes co-expressed in peanut roots with nine *AhZIP* genes, three *AhFRO* genes, two *AhNRAMP* genes, and two *AhOPT* genes, as well as *AhMTP9.1* and *AhNAS2.1* (Figure 7C).

### 2.7. Validation of DEG Results

**Figure 8:**
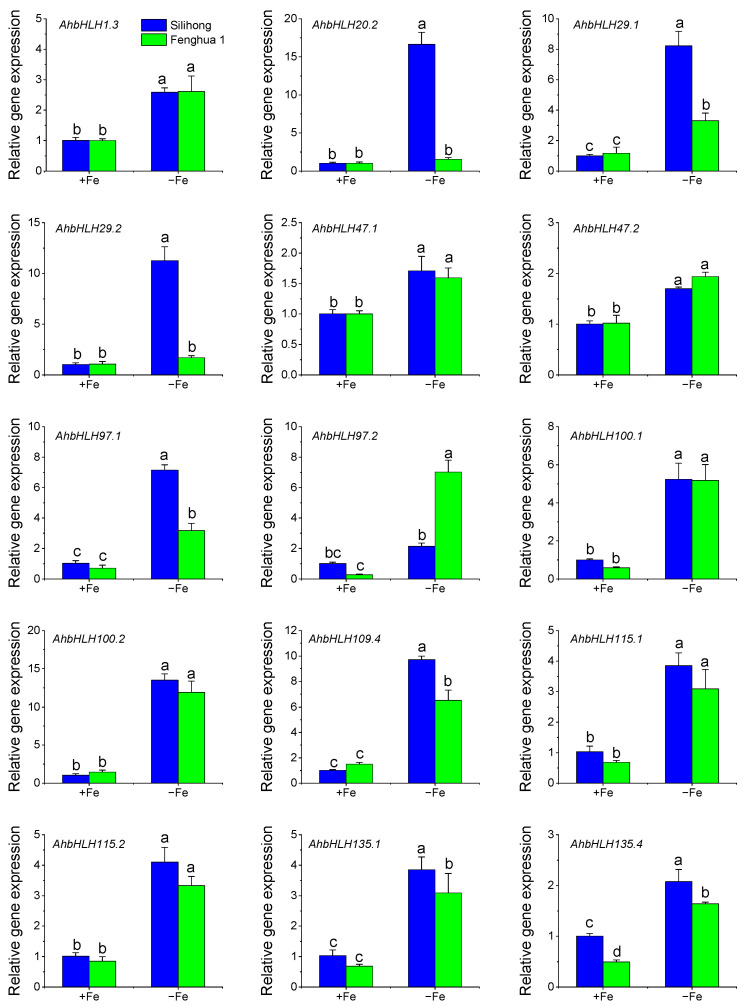
RT-qPCR validation of 15 selected *AhbHLH* genes in roots of Silihong and Fenghua 1 under Fe-sufficient (+Fe) and Fe-deficient (−Fe) conditions. Data (means ± SE, *n* = 3) sharing the same letter(s) above the error bars are not significantly different at the 0.05 level based on the Duncan multiple range test.

To validate the results of the RNA-seq-based comparative transcript analysis, 15 DEGs were selected for qRT-PCR analysis (Figure 8). The results showed that Fe deficiency significantly induced the expression of *AhbHLH001.3*, *AhbHLH029.1*, *AhbHLH047.1*/*.2*, *AhbHLH059.1*, *AhbHLH097.1*, *AhbHLH100.1*/*.2*, *AhbHLH109.4*, *AhbHLH115.1*/*.2*, and *AhbHLH135.1*/*.4* for both cultivars (Figure 8). The expression of *AhbHLH020.1* and *AhbHLH029.2* was upregulated by Fe deficiency in Silihong, but not in Fenghua 1. Compared with Fenghua 1, Silihong exhibited a higher expression of *AhbHLH020.1*, *AhbHLH029.1*/*.2*, *AhbHLH097.1*, *AhbHLH109.4*, and *AhbHLH135.4* under Fe-sufficient conditions (Figure 8). These data concurred with the RNA-seq results and indicated that the DEGs might be involved in the responses of peanut to Fe deficiency.

## 3. Discussion

### 3.1. WGD or Segmental Duplication Facilitates the Expansion of the bHLH Gene Family

In this study, we identified 373 *AhbHLH* genes from the peanut genome. The number of *AhbHLH* genes in peanut is more than that in *Arabidopsis* (162), *Brassica oleracea* (256) [30], *Raphanus sativus* (213) [31], tomato (159) [32], tobacco (309) [33], wheat (225) [34], maize (208) [35], poplar (202) [36], *Ziziphus jujuba* (92) [37], *Vernicia fordii* (104) [38], *Aquilaria sinensis* (105) [39], *Passiflora edulis* (138) [40], *Malus sieversii* (184) [41], *Artemisia annua* (226) [42], *Cymbidium ensifolium* (94) [43], and *Brachypodium distachyon* (146) [44], but less than that in cotton (*Gossypium hirsutum*, 437) [45], *Brassica napus* (602) [46], and *Phyllostachys pubescens* (448) [47]. Large numbers of genes have been reported in other families in peanut, such as metal tolerance proteins [48], natural resistance-associated macrophage proteins [49], oligopeptide transporters [50], and zinc/iron-regulated transporter-like proteins [51].

The expansion and functional diversification of gene families is facilitated by gene duplication, including WGD and segmental and tandem duplication [52]. Peanut, as an allotetraploid species, contains two sets of subgenomes (A and B) from two progenitors, *A. duranensis* (AA) and *A. ipaensis* (BB) [53], and has experienced at least three rounds of WGD events together with allopolyploidization [54]. In the current study, we found that almost all *AhbHLH*s are multicopy genes. Syntenic analysis revealed that 271 collinear blocks might be WGDs, and 108 collinear blocks might be segmental duplications, whereas only 10 collinear blocks were identified as tandem duplications. Therefore, large numbers of *AhbHLH* genes in peanut might be the result of WGD or segmental duplication. Similar results have been reported in tobacco [33].

The distribution of *Ks* values revealed two significant peaks in paralogous pairs (Figure 4B). The one near 0.0 represents the recent WGD from an allopolyploidization event, while the other smaller peaks near 1.0 might represent the latest ancient WGD event, from which the majority of segmental duplication occurred (Figure 4B). Similarly, the average *Ks* value of segmental duplicated gene pairs (1.25) is considerably higher than that of WGD (0.54). These data suggest that the *AhbHLH* gene family has expanded at least twice, including the recent allopolyploidization event.

### 3.2. Structural Characteristics of the bHLH Gene Family

Phylogenetic analysis revealed that the bHLH proteins can be divided into 14 groups (subfamilies). Although the number of protein sequences in our study is three times higher than in Heim et al. [28], the classification is identical except for three novel groups (Groups VI, XIII, and XIV). The current results support the classification of Heim et al. [28]. The clustering of bHLH proteins within these groups is further supported by structural characteristics such as conserved motif composition, conserved domains, exon/intron organization, and predicted DNA binding capacity. In many groups, proteins generally share similar conserved motif composition and conserved domains, showing similar predicted DNA binding capacity. As for gene structure, members within each group contain a similar intron number with conserved positions. These data support the general conclusion that members within groups or subgroups may have derived from common gene duplication events and, therefore, might have related molecular functions [3].

The bHLH proteins had a typical bHLH domain containing a basic region, two helices, and a loop connecting the helices [3,28]. The bHLH domain sequences of AhbHLH proteins contained ten residues with at least 70% conservation across all members, including four Leu (Leu-27, 43, 53, and 60) and three Arg (Arg-14, 16, and 17), as well as Glu-13, Pro-32, and Tyr-49. Glu-13 and Arg-16/Arg-17 in the basic region of the bHLH domain have been shown to play important roles in DNA binding [5], while Leu-27 and Leu-53 (and Leu-73 for Carretero-Paulet et al., 2010) in the helix regions participate in dimerization [55]. Therefore, these conserved residues lay the structural foundation for the molecular function of the bHLH protein.

The regulatory roles of bHLHs are based on the recognition of the core hexanucleotide sequence at the promoter of target genes, including the canonical E-box (CANNTG) and its variants (i.e., G-box, CACGTG), as well as non-E-box motifs such as the N-box variants (CACGGC and CACGAC) [3,55]. Key residues of the basic region play an important role in discriminating variants of the hexanucleotide core motif. Based on the presence of Glu-13 and Arg-16 in the basic region, 247 AhbHLH proteins (66.2%) were predicted to be E-box binders. Among them, 184 members were identified to be G-box binders according to the presence of His/Lys-9, Glu-13, and Arg-17 residues. In addition, 61 and 65 AhbHLH proteins were identified to be non-E-box binders (five to nine basic residues) and non-DNA binders (basic residues less than five), respectively. None of these DNA-binding categories formed monophyletic groups except Group XIII. These data support the idea that specific DNA-binding properties of the bHLH gene family might have evolved independently at different times [55].

### 3.3. AhbHLH Genes Play Regulatory Roles in Iron Homeostasis in Peanut

Iron deficiency is one of the main factors limiting the growth and development of peanut plants. To gain insights into the possible roles of AhbHLH genes in Fe deficiency responses, a comparative transcription analysis was performed on Fenghua 1 and Silihong under Fe-deficient or -sufficient conditions. A total of 55 DEGs were identified in the AhbHLH family. Among them, 19 DEGs were shared by at least two comparisons (Figure 6A). In terms of phylogeny, these DEGs are dispersed in eight different groups, such as Group I (*AhbHLH096.4*/*.8* and *AhbHLH097.1*/*.2*), III (*AhbHLH001.3*, *AhbHLH014.1*, and *AhbHLH029.1*/*.2*), IV(*AhbHLH020.2*, *AhbHLH041.2*, *AhbHLH047.1*/*.2*, and *AhbHLH115.1*/*.2*), VI (*AhbHLH147.2*), VII (*AhbHLH109.4*), VIII (*AhbHLH086.2*), and XIII (*AhbHLH135.1*/*.4*), indicating the independent acquisition of Fe deficiency responses at different evolutionary time.

The bHLH IVc subgroup has been showed to play crucial roles at the top of the regulatory network of Fe homeostasis in *Arabidopsis*. Members of the IVc subgroup can form homo- and heterodimers themselves or interact with bHLH121 and regulate the expression of bHLH Ib genes as well as *FIT* and *PYE* [11,12,13,14,15,16,17]. Unlike the IVc subgroup of *Arabidopsis* that contains four members, *bHLH34*/*104*/*105*/*115*, eight bHLH IVc members were identified in peanut, including two homologs of *AhbHLH104* (*AhbHLH104.1*/*.2*), four homologs of *AhbHLH105* (*AhbHLH105.1*/*.2*/*.3*/*.4*), and two homologs of *AhbHLH115* (*AhbHLH115.1*/*.2*). The expression of the two homologs of *AhbHLH115*, namely, *AhbHLH115.1*/*.2*, were induced by iron deficiency, while other genes were not differentially expressed in peanut roots. The WGCNA results indicated that *AhbHLH115.1*/*.2* exhibited a co-expression with Fe-transport-related genes in the blue module, which significantly and positively correlated with chlorophyll content. Our results are somewhat different from those obtained for *Arabidopsis*, which showed that the expression of *bHLH34*/*104*/*105*/115 is unaffected by Fe deficiency [11,13,56]. The results indicate a different mechanism of the responses to Fe deficiency between peanut and *Arabidopsis*, and *AhbHLH115* likely plays a crucial role in Fe deficiency responses in peanut.

In *Arabidopsis*, two regulatory networks, FIT and PYE, were found to regulate Fe deficiency responses [57]. FIT interacts with bHLH Ib members to form heterodimers that directly facilitate the activation of Fe-deficiency-responsive genes such as *FRO2* and *IRT1* [18,19,20]. In peanut, two homologs of *FIT* or *bHLH029*, namely, *AhbHLH029.1*/*.2*, were identified. With regard to the bHLH Ib subgroups such as *bHLH38/39/100/101*, only two homologs (*AhbHLH100.1*/*.2*) of *bHLH100* were identified. The expression of *AhbHLH029.1*/*.2* and *AhbHLH100.1*/*.2* was significantly upregulated by Fe deficiency in the roots of two peanut cultivars, at least in Silihong. Moreover, *AhbHLH029.1*/*.2* was shown to be co-expressed with Fe-transport-related genes according to the WGCNA. These data suggest that the FIT-bHLH100 network might be involved in Fe deficiency responses in peanut.

PYE is a transcriptional repressor that belongs to the bHLH IVb subgroup. PYE was shown to interact with bHLH104, bHLH105, and bHLH115 to form heterodimers, which can bind to the promoters of several Fe-responsive genes such as *NAS4* to repress their expression, consequently altering Fe translocation [11,12,21]. Our results revealed that the two homologs of *PYE* or *bHLH047*, namely, *AhbHLH047.1*/*.2*, show significantly higher expression in the Fe-deficient treatment for both cultivars compared with the control. The WGCNA results indicated that *AhbHLH047.1*/*.2* significantly and positively correlated with chlorophyll content, and were co-expressed with Fe-transport-related genes in the constructed gene network. These findings suggest that the PYE network might participate in the regulation of Fe homeostasis in peanut.

Moreover, the WGCNA results also revealed that several DEGs, namely, AhbHLH001.3, AhbHLH097.1/.2, AhbHLH109.4, and AhbHLH135.1, are significantly and positively correlated with chlorophyll content, and were co-expressed with Fe-transport-related genes in the constructed gene network, suggesting a potential role in Fe deficiency responses in peanut. Nine genes (AhbHLH001.3, AhbHLH029.1/.2, AhbHLH047.1/.2, AhbHLH097.1/.2, and AhbHLH115.1/.2) were specifically induced by Fe deficiency in Silihong, and their expression was higher in Silihong than in Fenghua 1. Conversely, four genes (AhbHLH014.1, AhbHLH041.2, and AhbHLH096.4/.8) were specifically repressed by Fe deficiency in Silihong, showing lower expression compared with Fenghua 1. These genes might be responsible for higher tolerance to Fe deficiency in Silihong.

## 4. Materials and Methods

### 4.1. Plant Materials, Treatments, and RT-qPCR Analysis

Two peanut cultivars differing in Fe deficiency tolerance, Fenghua 1 (sensitive cultivar) and Silihong (tolerant cultivar), were used for experiments [26,49,51]. The seeds were surface-sterilized with 5% NaClO solution for 1 min, presoaked in deionized water for 24 h, and then sown in acid-washed vermiculite for germination. Three-day-old seedlings with uniform size were transplanted to polyethylene pots and cultured in hydroponics as described previously [58]. Ten-day-old seedlings were treated with 0 (Fe deficiency, −Fe) or 50 μM Fe-EDTA (Fe-sufficiency, +Fe), respectively. One pot with three seedlings per cultivar served as a replication, and each treatment was replicated three times. Nutrient solutions were renewed twice a week. After seven days of treatment, fresh root tissues were sampled, immediately frozen in liquid nitrogen, and stored at −80 °C for RT-qPCR analysis.

Total RNA extraction, first-strand cDNA synthesis, and RT-qPCR analysis were performed as per the method described previously [49], with 60S ribosomal protein L7-2 (NCBI_ID: 112697914) as the internal control. The primers are listed in Table S7. Each sample was repeated with three technical replicates. The relative gene expression was calculated using the delta–delta CT method (2^−ΔΔCT^) [59].

### 4.2. Identification and Bioinformatics Analyses of bHLH Genes in Peanut

The sequences of 162 AtbHLH proteins (AtbHLH 1-162) were used as queries for TBLASTP against the genome of peanut (*A. hypogaea* cv. Tifrunner) using TBtools v. 1.112 software [60]. The Batch CD-Search tool (https://www.ncbi.nlm.nih.gov/Structure/bwrpsb/bwrpsb.cgi, accessed on 22 May 2023) was used to confirm the presence of the bHLH domain [61]. To further validate and filter out uncertain bHLH proteins, multiple sequence alignments were performed on candidate bHLHs using ClustalW in the MEGA-X program (v.10.2.6), according to the method described by Atchley et al. [62] and Toledo-Ortiz [3].

To investigate the phylogenetic relationships of the AhbHLHs, full-length sequences of bHLH proteins from peanut and *Arabidopsis* were used to construct a phylogenetic tree using the MEGA-X program (v.10.2.6), with the neighbor-joining method (p-distance) and 1000 bootstrap replicates. The phylogenetic tree was displayed and modified using iTOL (https://itol.embl.de/itol.cgi, accessed on 12 November 2023).

The physical and chemical properties of the AhbHLH proteins were analyzed using the ProtParam tool (https://web.expasy.org/protparam/, accessed on 20 November 2023). The conserved domains and motifs were analyzed using the Batch CD-Search tool (https://www.ncbi.nlm.nih.gov/Structure/bwrpsb/bwrpsb.cgi, accessed on 26 November 2023) and MEME v. 5.5.0 (https://meme-suite.org/meme/tools/meme, accessed on 28 November 2023), respectively [61,63]. The exon/intron structures were detected with genomic and coding sequences using GSDS v. 2.0 (http://gsds.gao-lab.org/, accessed on 10 December 2023) [64]. The synteny relationship of *AhbHLH* genes within peanut genome was analyzed using one-step MCScanX integrated in TBtools v. 1.112 [60]. *Ka*, *Ks*, and *Ka*/*Ks* were analyzed by simple *Ka*/*Ks* Calculator (NJ) in TBtools v. 1.112 [60].

### 4.3. Transcriptional Responses of AhbHLH Genes to Fe Deficiency

Transcription of *AhbHLH* genes in the roots of Fenghua 1 and Silihong in different Fe treatments were analyzed using RNA-seq data. The methods of plant culture, total RNA extraction, cDNA library construction, RNA sequencing, data filtering, and mapping are detailed in our previously published work [26]. Differentially expressed genes (DEGs) were detected using the DESeq2 R package (v.1.16.1). The determination criteria for DEGs are a fold change (FC) at least two times higher or lower (|log2FC| ≥ 1) and *p*-values adjusted by the Benjamini–Hochberg method (*P*_adj_) < 0.05. A heatmap diagram was constructed with lg^(TPM+1)^ using OriginPro 2021 (Originlab Corp., Northampton, MA, USA).

### 4.4. Co-Expression Analysis

Weighted gene co-expression networks were constructed based on a topology overlap matrix (TOM) using the WGCNA package in R (v.3.6.1) [65]. An appropriate soft threshold power *β* value was selected to be 16 according to the pick soft threshold function. The topological overlap matrix (TOM) was constructed for clustering and segmenting the modules. The correlation between module eigengene values (ME) and traits was calculated to identify the Fe-deficiency-related module. The exportNetworkToCytoscape function was used for exporting network edge and node information of modules. The network was visualized using Cytoscape (v. 3.9.1) [66].

### 4.5. Statistical Analysis

Data were subjected to one-way ANOVA using IBM SPSS Statistics v. 22 (IBM, New York, NY, USA), and significant differences among means were determined by the Duncan’s multiple-range test (*p* < 0.05).

## 5. Conclusions

In conclusion, a total of 373 *AhbHLH* genes were identified in peanut, which were divided into 14 groups or subfamilies. The majority of the AhbHLH proteins had a typical bHLH domain, while several phylogenetic groups including Group VI, X, XIII, and XIV had the HLH domain. Clustered *AhbHLH*s generally share similar gene/protein structures. Several *AhbHLH* genes including *AhbHLH001.3*, *AhbHLH029.1*/*.2*, *AhbHLH047.1*/*.2*, *AhbHLH115.1*/*.2*, *AhbHLH097.1*/*.2*, *AhbHLH109.4*, and *AhbHLH135.1* could play important roles in regulating iron homeostasis in peanut. The sensitivity of these genes to Fe deficiency and their higher expression under Fe-deficient conditions might be responsible for Fe deficiency tolerance in Silihong. These findings provide essential information and valuable clues for further elucidating the regulatory mechanism of Fe homeostasis in peanut. Unfortunately, none of *AhbHLH* genes have been functionally characterized in peanut. To fully understand the role of *AhbHLH* genes in the regulation of iron homeostasis in peanut, detailed functional characterization is required in future studies.

## Data Availability

RNA-seq clean reads are available from the NCBI Sequence Read Archive (SRA) database (https://www.ncbi.nlm.nih.gov/sra, accessed on 15 November 2023) under the BioProject Numbers PRJNA559452 (Fenghua 1) and PRJNA550213 (Silihong).

## Supplementary Materials

The following supporting information can be downloaded at https://www.mdpi.com/article/10.3390/ijms252212057/s1.
