# Supplementary material for: Genome-Wide Identification of Basic Helix–Loop–Helix (bHLH) Family in Peanut: Potential Regulatory Roles in Iron Homeostasis
Source: Int J Mol Sci. 2024 Nov 9;25(22):12057. doi: 10.3390/ijms252212057 (PMC11594023; doi:10.3390/ijms252212057)
Supplement: Supplementary file 1 [file ijms-25-12057-s001.zip › Supplementary Figure,.pdf]

```
AhbHLH001.1  EADENGMNHVLSER...RRRAKLN......ERFLTLRSMVPSI..........TKD................DKVSILDD..AIEYLRKLEKRIK........EL60
AhbHLH001.2  EVDDVGMNHALSER...KRRAKLN......ERFLTLRSMVPTI..........SKD................DKVSILDD..AMEYLRKLEEKVR........KL60
AhbHLH001.3  EADENGMNHVLSER...RRRAKLN......ERFLTLRSMVPSI..........TKD................DKVSILDD..AIEYLRKLEKRIK........EL60
AhbHLH001.4  EVDDVGMNHALSER...KRRAKLN......ERFLTLRSMVPTI..........SKD................DKVSILDD..AMEYLRKLEEKVR........KL60
AhbHLH003.1  NGREEPLNHVEAER...QRREKLN......QRFYALRAVVPNI..........SKM................DKASLLGD..AITYITDLQMKIK........VL60
AhbHLH003.2  NGREEPLNHVEAER...QRREKLN......QRFYALRAVVPNI..........SKM................DKASLLGD..AITYITDLQMKIK........VL60
AhbHLH003.3  NGKEEPLNHVEAER...QRRKKLN......QWFYALRAVVPNI..........SKM................DRASLLGD..EQPVVAKGVEE............56
AhbHLH004.1  .GREEPLNHVEAER...QRREKLN......QRFYALRAVVPNV..........SKM................DKASLLGD..AISYINELKSKLQ........GL59
AhbHLH004.2  .GREEPLNHVEAER...QRREKLN......QRFYALRAVVPNV..........SKM................DKASLLGD..AISYITELKSKLQ........TL59
AhbHLH004.3  .GREEPLNHVEAER...QRREKLN......QRFYALRAVVPNV..........SKM................DKASLLGD..AISYINELKSKLQ........GL59
AhbHLH004.4  .GREEPLNHVEAER...QRREKLN......QRFYALRAVVPNV..........SKM................DKASLLGD..AISYITELKSKLQ........TL59
AhbHLH008.1  KRSRSAEVHNLSER...RRRDRIN......EKMRALQDLIP...........NCN...............KVDKASMLDE..AIEYLKTLQLQVQ........IF60
AhbHLH008.2  KRSRAAEVHNLSER...RRRDRIN......EKMRALQELIP...........NCN...............KVDKASMLDE..AIEYLKTLQLQVQ........MM60
AhbHLH008.3  KRSRSAEVHNLSER...RRRDRIN......EKMRALQDLIP...........NCN...............KVDKASMLDE..AIEYLKTLQLQVQ........IM60
AhbHLH008.4  KRSRAAEVHNLSER...RRRDRIN......EKMRALQELIP...........NCN...............KVDKASMLDE..AIEYLKTLQLQVQ........MM60
AhbHLH009.1  RRNRAAEVHNLSER...RRRDRIN......EKMRTLQQLIP...........NSN...............KTDKASMLEE..AIEYLKSLQLQLQ........VM60
AhbHLH009.2  RRNRAAEVHNLSER...RRRDRIN......EKMRTLQQLIP...........NSN...............KTDKASMLEE..AIEYLKSLQLQLQ........VM60
AhbHLH012.1  TKEWTNRLENADDK...FRENVLSDKIRETGNYKILKSSIPHPI.........SEV.................EKISILGD..TIKYLKELETRVE........EL67
AhbHLH012.2  TKEWTNRLENADDK...FRENVLSDKIRETGNFKILKSSIPHPI.........SEV.................EKISILGD..TIKYLKELETRVE........EL67
AhbHLH013.1  NGREEPLNHVEAER...QRREKLN......QRFYALRSVVPNI..........SKM................DKASLLGD..AIAYINELQAKLK........MM60
AhbHLH013.2  NGREEPLNHVEAER...QRREKLN......QRFYALRSVVPNI..........SKM................DKASLLGD..AIAYINELQAKLK........MM60
AhbHLH014.1  .NRETPVNHVEAER...QRREKLN......HRFYALRAVVPNV..........SRM................DKASLLSD..AVAYINELKAKIK........DL59
AhbHLH014.2  .GRETPLNHVEAER...QRREKLN......HRFYALRAVVPNV..........SRM................DKASLLSD..AVSYINELKAKIE........EL59
AhbHLH014.3  .NRETPVNHVEAER...QRREKLN......HRFYALRAVVPNV..........SRM................DKASLLSD..AVAYINELKAKIK........DL59
AhbHLH014.4  .GRETPLNHVEAER...QRREKLN......HRFYALRAVVPNV..........SRM................DKASLLSD..AVSYINELKAKIE........EL59
AhbHLH015    KRSRAAEVHNLSER...RRRDRIN......ERMRALQELIL...........RCN...............KSDKASMLDE..AIEYLKSLKLQVQ........MM60
AhbHLH016.1  KRSRAAAIHNQSER...KRRDKIN......QRMKTLQKLVP...........NSS...............KTDKASMLDE..VIEYLKQLQAQVQ........MV60
AhbHLH016.2  KRSRAAAIHNQSER...KRRDKIN......QRMKTFQKLVP...........NSS...............KTDKASMLDE..VIEYLKQLQAQVQ........MV60
AhbHLH018.1  SRLSQPQDHIIAER...KRREKLS......QRFIALSALVP...........GLQK...............TDKASVLGD..AIKYLKQLQEKVK........AL60
AhbHLH018.2  LSSHQPHDHIIAER...KRREKLS......QRFIALSALVP...........GLKK...............MDKASVLGD..AIKYLKQMQEKVS........AL60
AhbHLH018.3  SRLSQPQDHIIAER...KRREKLS......QRFIALSALVP...........GLQK...............TDKASVLGD..AIKYLKQLQEKVK........AL60
AhbHLH018.4  LSSHQPHDHIIAER...KRREKLS......QRFIALSALVP...........GLKK...............MDKASVLGD..AIKYLKQMQEKVS........AL60
AhbHLH019.1  RSPSHAHDHIIAER...KRREKIS......QSFIALAALVP...........GLKK...............MDKASVLGD..SIRYVKELKERLA........VL60
AhbHLH019.2  RSPSNAQDHIIAER...KRREKIS......QSFIALAALVP...........GLKK...............IDKVTVLEN..AIKYVKDLKIRLT........TL60
AhbHLH019.3  RQAAHSHDHIMAER...KRREKLS......QSLIALAALIP...........GLKK...............MDKASVLGD..AIKYVKELQGRLK........VL60
AhbHLH019.4  RSPQHAHDHIIAER...MRREKIS......QQFIALSALIP...........GLKK...............MDKATVLGD..AIKYVKQLQEQVK........VL60
AhbHLH019.5  RSPSNAQDHIIAER...KRREKIS......QSFIALAALVP...........GLKK...............IDKVTVLEN..AIKYVKDLKTRLT........TL60
AhbHLH019.6  RSPSHAHDHIIAER...KRREKIS......QSFIALAALVP...........GLKK...............MDKASVLGD..SIKYVKELKERLA........VL60
AhbHLH019.7  RQAAHSHDHIMAER...KRREKLS......QSLIALAALIP...........GLKK...............MDKASVLGD..AIKYVKELQGRLK........VL60
AhbHLH019.8  RSPQHAHDHIIAER...MRREKIS......QQFIALSALIP...........GLKK...............MDKATVLGD..AIKYVKQLQEQVK........VL60
AhbHLH020.1  KRKGKTVDHIIAER...KRRQDLT......RSIIQLSATIP...........GLKK...............MDKAHVIRE..SLSYIKILQDRVK........EL60
AhbHLH020.10 RNDSQIVDHIMAER...KRRQQLT......QMFIALSATIP...........GLKK...............TDKASILGE..AINYVKQLQERVR........EL60
AhbHLH020.11 RSASETADHIMSER...NRRQELS......RKFIALSATIP...........GLK...................KAHVLEE..AIKYVKELEERVK........VL57
AhbHLH020.12 RSSSECLDHIVAER...KRRQEIT......QRFIALSATIP...........HLKK...............IDKVSILSE..AIAYIKQLKEQGK........KL60
AhbHLH020.13 RSSSETLDHIMTER...KRRRELT......ERFIALSATIP...........GLKK...............IDKSTILSE..AISHVKQLKQRVK........EL60
AhbHLH020.14 EEALDPIMDETQRK...KKQRELE......ERLLALSATIP...........SSGFKN...........NKMDDRSILDN..ASNYVKQLQQRVR........EL64
AhbHLH020.15 RSSSQTMDHIMAER...KRRQELT......ERFIALSATIP...........GLTK...............TDKASILRA..AIDYVKQLQERVQ........EL60
AhbHLH020.16 RNSIEALDHIMSER...KRRQQMA......EKFIALAAAIP...........GLKK...............IDKASILQE..AINYVKELQERIA........LL60
AhbHLH020.2  RNDSQIVDHIMAER...KRRQQLT......QMFIALSATIP...........GLKK...............TDKASILGE..AINYVKQLQERVR........EL60
AhbHLH020.3  RSASETADHIMSER...NRRQELS......RKFIALSATIP...........GLK...................KAHVLEE..AIKYVKELEERVK........VL57
AhbHLH020.4  RSSSECLDHIVAER...KRRQEIT......QRFIALSATIP...........HLKK...............IDKVSILSE..AIAYIKQLKEQGK........KL60
AhbHLH020.5  RSSSQTMDHIMAER...KRRQELT......ERFIALSATIP...........GLTK...............TDKASILRA..AIDYVKQLQEQVQ........EL60
AhbHLH020.6  RNSIEALDHIMSER...KRRQQMA......EKFIALAAAIP...........GLKK...............IDKASILQE..AINYVKELQERIA........LL60
AhbHLH020.7  RSSSETVDHIMTER...KRRRELT......ERFIALSATIP...........GLKK...............IDKSTILSE..AISHVKQLKQRVK........EL60
AhbHLH020.8  EEALDPIMDETQRK...KKQRELE......ERLLALSATIP...........SSGFKN...........NKMDDRSILDN..ASNYVKQLQQRVR........EL64
AhbHLH020.9  KRKGKTADHIIAER...KRRQDLT......RSIIQLSATIP...........GLKK...............MDKAHVIRE..SLSYIKVLQDRVK........EL60
AhbHLH021.1  GKGNQS.KNLVAER...KRRKKLN......DRLYNLRSLVP...........RISK...............LDRASILGD..AIEFVKDLQKQVK........DL59
AhbHLH021.2  GKGNQS.KNLVAER...KRRKKLN......DRLYNLRSLVP...........RISK...............LDRASILGD..AIEFVKDLQKQVK........DL59
AhbHLH022    TVVYKS.KNLETER...RRRQKLS......DRLLMLR.................SL...............MNKATIIED..AITYIKNLKDKVD........SL53
AhbHLH024.1  KRSRAAEVHNLSEK...RRRSRIN......EKMKALQNLIP...........NSN...............KTDKASMLDE..AIEYLKQLQLQVQGKFMIIFQML68
AhbHLH024.2  KRSRAAEFHNLSEK...RRRSRIN......EKMKALQNLIP...........NSN...............KTDKASMLDE..AIEYLKQLQLQVQ........ML60
AhbHLH024.3  KRSRAAEVHNLSEK...RRRSRIN......EKMKALQNLIP...........NSN...............KTDKASMLDE..AIEYLKQLQLQVQGKFMIIFQML68
AhbHLH024.4  KRSRAAEFHNLSEK...RRRSRIN......EKMKALQNLIP...........NSN...............KTDKASMLDE..AIEYLKQLQLQVQ........ML60
AhbHLH025.1  RNPTQAQDHVIAER...KRREKLS......QRFIALSAIVP...........GLKK...............MDKASVLGD..AIKYLKQLQEKVK........IL60
AhbHLH025.2  RNPIQAQEHVIAER...KRREKLS......QRFVALSAMVP...........GLKK...............MDKASILGD..AIKYVKQLQERVQ........FL60
AhbHLH025.3  RNPTQAQDHVIAER...KRREKLS......QRFIALSAIVP...........GLKK...............MDKASVLGD..AIKYLKQLQEKVK........IL60
AhbHLH025.4  RNPIQAQEHVIAER...KRREKLS......QRFVALSAMVP...........GLKK...............MDKASILGD..AIKYVKQLQERVQ........FL60
AhbHLH029.1  LKADRS.KTLISER...RRRSRMK......EKLYALRSLVP...........NITK...............MDKASIIGD..ALSYVHELQAQAK........KL59
AhbHLH029.2  VKADRS.KTLISER...RRRSRMK......EKLYALRSLVP...........NITK...............MDKASIIGD..ALSYVHELQAQAK........KL59
AhbHLH030.1  KALAASKSHSEAER...RRRERIN......NHLAKLRSLLP...........NTT...............KTDKASLLAE..VIQHVKELKRQTS........LI60
AhbHLH030.2  KALAASKSHSEAER...RRRERIN......NHLSKLRSLLP...........STT...............KTDKASLLAE..VIQHVKELKRQTS........MI60
AhbHLH030.3  KALAASKSHSEAER...RRRERIN......NHLSKLRSLLP...........STT...............KTDKASLLAE..VIQHVKELKRQTS........MI60
AhbHLH030.4  KALAASKSHSEAER...RRRERIN......NHLSKLRSLLP...........STT...............KTDKASLLAE..VIQHVKELKRQTS........MI60
AhbHLH030.5  KALAASKSHSEAER...RRRERIN......NHLSKLRSLLP...........STT...............KTDKASLLAE..VIQHVKELKRQTS........MI60
AhbHLH030.6  KALAASKSHSEAER...RRRERIN......NHLAKLRSLLP...........NTT...............KTDKASLLAE..VIQHVKELKRQTS........LI60
AhbHLH030.7  KALAASKSHSEAER...RRRERIN......NHLSKLRSLLP...........STT...............KTDKASLLAE..VIQHVKELKRQTL........MI60
AhbHLH031.1  RRGQATDSHSLAER...ARREKIS......ERMKILQDLVPG.........CNK...............VIGKALVLDE..IINYIQSLQRQVE........FL61
AhbHLH031.2  RRGQATDSHSLAER...ARREKIS......ERMKILQDLVPG.........CNK...............VIGKALVLDE..IINYIQSLQRQVE........FL61
AhbHLH031.3  RRGQATDSHSLAER...ARREKIS......ERMKILQDLVPG.........CNK...............VIGKALVLDE..IINYIQSLQRQVE........FL61
AhbHLH031.4  RRGQATDSHSLAER...ARREKIS......ERMKILQDLVPG.........CNK...............VIGKALVLDE..IINYIQSLQRQVE........FL61
AhbHLH031.5  ....MVSTSSANDL...ARREKIN......ERMKILQDLVPG.........CNK...............VIGKALVLDK..IINYIQSLQRQVE........FL57
AhbHLH033.1  KTGMPA.KNLMAER...RRRKKLN......DRLYMLRSVVP...........NISK...............MDRASILGD..AIEYMKELLQKIN........DL59
AhbHLH033.2  KKGMPA.KNLMAER...RRRKKLN......DRLYMLRSVVP...........KISK...............MDRASILGD..AIEYLRELLQRIN........EL59
AhbHLH033.3  KTGMPA.KNLMAER...RRRKKLN......DRLYMLRSVVP...........NISK...............MDRASILGD..AIEYMKELLQKIN........DL59
AhbHLH033.4  KKGMPA.KNLMAER...RRRKKLN......DRLYMLRSVVP...........KISK...............MDRASILGD..AIEYLRELLQRIN........EL59
AhbHLH035.1  ASSAPSNKNIVSER...NRRKKLN......ERLFALRAVVP...........NITK...............MDKASIIKD..AIDYIEHLHEQEK........RI60
AhbHLH035.2  ASSAAS.KNIVSER...NRRKKLN......ERLFALRSVVP...........NISK...............MDKASIIKD..AIEYIQKLQEQER........II59
AhbHLH035.3  ASSAAS.KNIVSER...NRRKKLN......ERLFALRAVVP...........NITK...............MDKASIIKD..AIDYIEHLHEQER........RI59
AhbHLH035.4  ASSAAS.KNIVSER...NRRKKLN......ERLFALRSVVP...........NISK...............MDKASIIKD..AIEYIQKLQEQER........II59
AhbHLH036.1  DNKKKKMVHREIER...QRRKEMA......TLYSSLRSLLP........LQFIKGK...............RSISDHMNE..AVNYIKYMQNNIK........EL63
AhbHLH036.2  .EENKKWMHRETER...QRRQEMG......KLCATLRSLLP........LEYIKGK...............RSTSDHMYE..AVNYINQLQNKVK........KL62
AhbHLH036.3  GDENKKWMHREIEK...QRRQEMS......RLCTTFRTLLP........FEYIKGK...............RSTSDHMHE..GVKYIKHLQNRVE........KL63
AhbHLH036.4  DNNKKKMVHREIER...QRRKEMA......TLYSSLRSLLP........LQFIKGK...............RSISDHMNE..AVNYIKYMQNNIK........EL63
AhbHLH036.5  TEENKKWMHRETEK...QRRQEMG......KLCATLRSLLP........LEYIKGK...............RSTSDHMYE..AVNYINQLQNKVK........KL63
AhbHLH036.6  GDENKKWMHREIEK...QRRQEMS......RLCTTFRTLLP........FEYIKGK...............RSTSDHMHE..GVKYIKHLQNRVE........KL63
AhbHLH037.1  NVKISKDPQSVAAR...HRRERIS......EKIRILQRLVPG.........GTK...............MDTASMLDE..AVHYVKFLKKQVQ........TL60
AhbHLH037.2  NVKISKDPQSVAAR...HRRERIS......ERIRILQRLVPG.........GTK...............MDTASMLDE..AIHYVKFLKKQVQ........TL60
AhbHLH037.3  NVKISKDPQSVAAR...HRRERIS......ERIRILQRLVPG.........GTK...............MDTASMLDE..AIHYVKFLKAQIH........SL60
AhbHLH037.4  NVKISKDPQSVAAR...HRRERIS......EKIRILQRLVPG.........GTK...............MDTASMLDE..AVHYVKFLKKQVQ........TL60
AhbHLH037.5  .MKLSTDPQSVAAR...ERRHRIS......DRFKILQSMVPG.........GSK...............MDTVSMLEE..AIHYVKFLKAQIW........LH59
AhbHLH037.6  NVKISKDPQSVAAR...HRRERIS......ERIRILQRLVPG.........GTK...............MDTASMLDE..AIHYVKFLKKQVQ........TL60
AhbHLH037.7  NVKISKDPQSVAAR...HRRERIS......ERIRILQRLVPG.........GTK...............MDTASMLDE..AIHYVKFLKAQIH........SL60
AhbHLH041.1  ANNNKHLHHIILER...RRRENEN......KCFRELRALLPPG..........TKK................HKSSVLIA..AKEALKSLIAEIE........KL60
AhbHLH041.2  RPSSTQLHHMIAER...RRREKLN......DNFQALRALLPPG..........TKK................DKASILTT..AKETLSSLMAEIE........KL60
AhbHLH041.3  ANNNKHLRHIILER...RRRENEN......KCFRELRALLPPG..........TKK................HKSSVLTA..AKEALKSLIAEIE........KL60
AhbHLH042.1  PQDDLISSHVAAER...RRREKLN......EKFITLRSLVPYV..........TKM................DKASILGD..TIEYVKQLHEKVK........KL60
AhbHLH042.2  PQDELSANHVLAER...RRREKLN......ERFIILRSLVPFV..........TKM................DKASILGD..TIEYVKQLRRKIQ........DL60
AhbHLH042.3  PQDDLISSHVAAER...RRREKLN......EKFITLRSLVPYV..........TKM................DKASILGD..TIEYVKQLHEKVK........KL60
AhbHLH043.1  NVRISDDPQSVAAR...HRRERIS......EKIRILQRLVPG.........GTK...............MDTASMLDE..AIRYVKFLKRQIR........LL60
```

```
AhbHLH043.2  NVRISDDPQSVAAR...HRRERIS......ERIRILQRLVPG.........GTK.................MDTASMLDE..AVRYVKFLKRQVR........FL60
AhbHLH043.3  NVRISDDPQSVAAR...HRRERIS......EKIRILQRLVPG.........GTK.................MDTASMLDE..AIRYVKFLKRQIR........LL60
AhbHLH043.4  NVRISDDPQSVAAR...HRRERIS......ERIRILQRLVPG.........GTK.................MDTASMLDE..AVRYVKFLKRQIR........FL60
AhbHLH044.1  RRGQATDTHSLAER...VRRGKIN......EKLKFLQNIVPG.........CYK................TMGMAIMLEE..IINYVQSLQHQVE........FL61
AhbHLH044.2  RRGQATDSHSIAER...VRRGKIN......EKLRCLQNIVPG.........CYK................TMGMAVMLDE..IINYVQSLQHQVE........FL61
AhbHLH044.3  RRGQATDTHSLAER...VRRGKIN......EKLKFLQNIVPG.........CYK................TMGMAIMLEE..IINYVQSLQHQVE........FL61
AhbHLH044.4  RRGQATDSHSIAER...VRRGKIN......EKLRCLQNIVPG.........CYK................TMGMAVMLDE..IINYVQSLQHQVE........FL61
AhbHLH045.1  ......MSHIAVER...NRRKQMN......EHLKVLRSLTPS........FYIKR................GDQASIIGG..VIEFIKELHQVLQ........AL56
AhbHLH045.2  ......MSHIAVER...NRRKQMN......EHLKVLRSLTPS........FYIKR................GDQASIIGG..VIEFIKELHQVLQ........AL56
AhbHLH046.1  KPNTPRSKHSATEQ...RRRSKIN......DRFQMLRELIP..........HSDQ...............KRDKASFLLE..VIEYIHFLQEKVH........KY61
AhbHLH046.2  KPNTPRSKHSATEQ...RRRSKIN......DRFQMLRELIP..........HSDQ...............KRDKASFLLE..VIEYIHFLQEKVH........KY61
AhbHLH047.1  GKVPKRIHKAEREK...MKREHLN......ELFLDLANVLDLN........EQNN.................GKASILNE..TARLLKDLLCQIE........SL61
AhbHLH047.2  GKVPKRIHKAEREK...MKREHLN......ELFLDLANVLDLN........EQNN.................GKASILNE..TARLLKDLLCQIE........SL61
AhbHLH048.1  RRGQATDSHSLAERVN.ARREKIN......ARMKLLQELVPG.........CNKERTKDIRYMRCNRTVGISGTALVLDE..IINHVQSLQRQVE........FL79
AhbHLH048.2  RRGQATDSHSLAER...ARREKIN......ARMKLLQELVPG.........CNK................ISGTALVLDE..IINHVQSLQRQVE........FL61
AhbHLH048.3  RRGQATDSHSLAERVN.ARREKIN......ARMKLLQELVPG.........CNKERTKDIRYMRCNRTVGISGTALVLDE..IINHVQSLQRQVE........FL79
AhbHLH048.4  RRGQATDSHSLAER...ARREKIN......ARMKLLQELVPG.........CNK................ISGTALVLDE..IINHVQSLQRQVE........FL61
AhbHLH049.1  RRGQATNSHSLAER...VRREKIS......ERMKFLQDLVPG.........CSK................VTGKAVMLDE..IINYVQSLQRQVE........FL61
AhbHLH049.2  RRGQATNSHSLAER...VRREKIS......ERMKFLQDLVPG.........CSK................VTGKAVMLDE..IINYVQSLQRQVE........FL61
AhbHLH049.3  RRGQATNSHSLAER...VRREKIS......ERMKFLQDLVPG.........CSK................VTGKAVMLDE..IINYVQSLQRQVE........FL61
AhbHLH049.4  RRGQATNSHSLAER...VRREKIS......ERMKFLQDLVPG.........CSK................VTGKAVMLDE..IINYVQSLQRQVE........FL61
AhbHLH051.1  RAASASKSHSQAEK...RRRDRIN......AQLATLRKLIP..........KSD................KMDKAALLGN..VVYHVKDLKRKAM........DV60
AhbHLH051.2  RAASASKSHSQAEK...RRRDRIN......AQLATLRKLIP..........KSD................KMDKAALLGS..VVDHVKDLKRKAM........DV60
AhbHLH051.3  RAASASKSHSQAEK...RRRDRIN......AQLTSLRKLIP..........KSD................KMDKAALLGS..VIEQVKDLKRKAI........EV60
AhbHLH051.4  RAASASKSHSQAEK...RRRDRIN......AQLATLRKLIP..........KSD................KMDKAALLGS..VVDHVKDLKRKAM........DV60
AhbHLH051.5  RAASASKSHSQAEK...RRRDRIN......AQLTSLRKLIP..........KSD................KMDKAALLGS..VIEQVKDLKRKAI........EV60
AhbHLH053.1  ...RTISAQSIAAR...ERRRKIT......EKTQELGKLVPG.........GPK.................MNTAEMLHA..ASRYVKYLQTQVG........ML57
AhbHLH053.2  DSERPISAQSIAAR...ERRKKIT......DKTQEFGKLVPG.........GPK.................MNTAEMLYA..ASKYVKYLQTQIG........ML60
AhbHLH053.3  LRAKPLPPKSNFAR...QRRQKLS......EKTRCLQKLMPW.........DKK.................MDQATLFEQ..AYSYVKFLQAQVS........VL60
AhbHLH053.4  ...RTISAQSIAAR...ERRRRIT......EKTQELGKLVPG.........GPK.................MNTAEMLHA..ASRYVKYLQTQVG........ML57
AhbHLH053.5  LRAKPLPPKSNFAR...QRRQKLS......EKTRCLQKLMPW.........DKK.................MDQATLFEQ..AYSYVKFLQAQVS........VL60
AhbHLH053.6  ...RTISAQSIAAR...ERRRNIT......EKTQELGKLVPG.........SSK.................MNTAEMLHA..ASKYVKYLQTQIG........ML57
AhbHLH054.1  IRGTAADPQSLYAR...KRRERIN......ARLRILQSLVPN.........GTK.................VDISTMLEE..AVNYVKFLQLQIK........LL60
AhbHLH054.2  TRGSATDPQSLYAR...RRRERIN......ERLRTLQSLVPN.........GTK.................VDISTMLEE..AVHYVKFLQLQIK........LL60
AhbHLH054.3  IRGTAADPQSLYAR...KRRERIN......ARLRILQSLVPN.........GTK.................VDISTMLEE..AVNYVKFLQLQIK........LL60
AhbHLH054.4  TRGSATDPQSLYAR...RRRERIN......ERLRTLQSLVPN.........GTK.................VDISTMLKE..AVHYVKFLQLQIK........LL60
AhbHLH057.1  DVENQRMTHIAVER...NRRRQMN......DHLSVLRSLMPP........SYIQR................GDQASIIGG..AIDFVKELEQLLQ........SL62
AhbHLH057.2  EVENQRMTHIAVER...NRRRQMN......EHLSVLKSLMHP........SYIQR................GDHASIIGG..AIDFVKELEQLVE........SL62
AhbHLH057.3  DVENQRMTHIAVER...NRRRQMN......DHLSVLRSLMPP........SYIQR................GDQASIIGG..AIDFVKELEQLLQ........SL62
AhbHLH057.4  EVENQRMTHIAVER...NRRRQMN......EHLSVLKSLMHP........SYIQR................GDHASIIGG..AIDFVKELEQLVE........TL62
AhbHLH059.1  RRGQATDPHSIAER...LRRERIA......ERIRALQELVPS.........VNK.................TDRAAMLDE..IVDYVKFLRLQVK........VL60
AhbHLH059.2  RRGQATDPHSIAER...LRRERIA......ERMKALQELVPS.........INK.................TDRAAMLDE..IVDYVKFLRLQVK........VL60
AhbHLH059.3  RRGQATDPHSIAER...LRRERIA......ERIRALQELVPS.........VNK.................TDRAAMLDE..IVDYVKFLRLQVK........VL60
AhbHLH059.4  RRGQATDPHSIAER...LRKERIA......ERIRALQELVSS.........VNK.................TDRTVMLDE..IIDLHLFISDTAL........I*59
AhbHLH059.5  RRGQATDPHSIAER...LRRERIA......ERIRALQELVPS.........VNK.................TDRAAMLDE..IVDYVKFLRLQVK........VL60
AhbHLH059.6  RRGQATDPHSIAER...LRRERIA......ERMKALQELVPS.........INK.................TDRAAMLDE..IVDYVKFLRLQVK........VL60
AhbHLH061.1  LEGQPS.KNLMAER...RRRKRLN......DRLSMLRSIVP..........KISK................MDRTSILGD..TIDYMKELLERIN........KL59
AhbHLH061.2  LEGQPS.KNLMAER...RRRKRLN......DRLSMLRSIVP..........KISK................MDRTSILGD..TIDYMKELLERIA........KL59
AhbHLH061.3  VEGQPS.KNLMAER...RRRKRLN......DRLSMLRSIVP..........KISK................MDRTSILGD..TIDYMKELLDKIN........TL59
AhbHLH061.4  VVGQPS.KNLMAER...RRRKRLN......DRLSMLRAIVP..........KISK................MDRTSILRD..TIDYIKELLEKIN........NL59
AhbHLH061.5  LEGQPS.KNLMAER...RRRKRLN......DRLSMLRSIVP..........KISK................MDRTSILGD..TIDYMKELLERIN........KL59
AhbHLH061.6  LEGQPS.KNLMAER...RRRKRLN......DRLSMLRSIVP..........KISK................MDRTSILGD..TIDYMKELLERIA........KL59
AhbHLH061.7  VEGQPS.KNLMAER...RRRKRLN......DRLSMLRSIVP..........KISK................MDRTSILGD..TIDYMKELLDKIN........TL59
AhbHLH061.8  VVGQPS.KNLMAER...RRRKRLN......DRLSMLRAIVP..........KISK................MDRTSILGD..TIDYMKELLEKIN........NL59
AhbHLH063.1  RRGQATDSHSLAER...VRREKIS......ERMKYLQDLVPG.........CNK................ITGKAGMLDE..IINYVQSLQRQVE........FL61
AhbHLH063.2  RRGQATDSHSLAER...VRREKIS......ERMKFLQDLVPG.........CNK................ITGKAGMLDE..IINYVQSLQRQVE........FL61
AhbHLH063.3  RRGQATDSHSLAER...VRREKIS......ERMKYLQDLVPG.........CNK................ITGKAGMLDE..IINYVQSLQRQVE........FL61
AhbHLH063.4  RRGQATDSHSLAER...VRREKIS......ERMKFLQDLVPG.........CNK................ITGKAGMLDE..IINYVQSLQRQVE........FL61
AhbHLH068.1  ARVQQS.TSSSTFK...VRKEKLG......DRIAALHQLVSP.........FGK.................TDTASVLLE..AIGYIRFLQSQIE........AL59
AhbHLH068.2  ARVQPSTTTQSTFK...VRKEKLG......DRITALHQLVSP.........FGK.................TDTASVLLE..AIGYIRFLQSQIE........AL60
AhbHLH068.3  ARVQ.....QSTFK...VRKEKLG......DRIAALHQLVSP.........FGK.................TDTASVLLE..AIGYIRFLQSQIE........AL55
AhbHLH068.4  ARVQPSTTTQSTFK...VRKEKLG......DRITALHQLVSP.........FGK.................TDTASVLLE..AIGYIRFLQSQIE........AL60
AhbHLH071.1  EAENQRMIHITVER...NRRKLMN......EHLAVLRSLMPE........SYVQR................GDQASIVGG..AIEFVKELEQLLQ........CL62
AhbHLH071.2  EAENQRMIHITVER...NRRKLMN......EHLAVLRSLMPE........SYVQR................GDQASIVGG..AIEFVKELEQLLQ........CL62
AhbHLH072.1  RRNRTAAVHNQSER...RRRDRIN......QKMKALQRLVP..........NAN................KTDKASMLDE..VIKYLKQLQAQIE........MM60
AhbHLH072.2  RRNRTAAVHNQSER...RRRDRIN......QKMKALQRLVP..........NAN................KTDKASMLDE..VIKYLKQLQAQIE........MM60
AhbHLH074.1  RRGQATNSHSLAER...VRREKIS......ERMRLLQELVPG.........CNK................ITGKAVMLDE..IINYVQSLQQQVE........FL61
AhbHLH074.2  RRGQATNSHSLAER...VRREKIS......ERMRLLQELVPG.........CNK................ITGKAVMLDE..IINYVQSLQQQVE........FL61
AhbHLH074.3  RRGQATNSHSLAER...VRREKIS......ERMRLLQELVPG.........CNK................ITGKAVMLDE..IINYVQSLQQQVE........FL61
AhbHLH074.4  RRGQATNSHSLAER...VRREKIS......ERMRLLQELVPG.........CNK................ITGKAVMLDE..IINYVQSLQQQVE........FL61
AhbHLH078.1  RRGQATDSHSLAER...VRREKIS......ERMKLLQDLVPG.........CNK................VTGKALMLDE..IINYVQSLQRQVE........FL61
AhbHLH078.2  RRGQATDSHSLAER...VRREKIS......ERMKLLQDLVPG.........CNK................VTGKALMLDE..IINYVQSLQRQVE........FL61
AhbHLH080.1  KRGFATHPRSIAER...VRRTRIS......ERIKKLQGLFPK.........SDKQ................TSTAEMLDL..AVEHIKELQEQVK........ML61
AhbHLH080.2  KRGFATHPRSIAER...ERRTRIS......ARIKKLQDLFPK.........SDKQ................TSTADMLDL..AVDYIKGLQKQVK........IL61
AhbHLH080.3  KRGFATHPRSIAER...VRRTRIS......ERIKKLQGLFPK.........SDKQ................TSTAEMLDL..AVEHIKELQEQVK........ML61
AhbHLH080.4  KRGFATHPRSIAER...ERRTRIS......ARIKKLQDLFPK.........SDKQ................TSTADMLDL..AVDYIKGLQKQVK........IL61
AhbHLH081.1  KRGCATHPRSIAER...VRRTRIS......DRIRKLQELVPN.........MDKQ................TNTADMLDE..AVAYVKSLQKQIE........EL61
AhbHLH081.2  KRGCATHPRSIAER...VRRTRIS......DRIRKLQELVPN.........MDKQ................TNTADMLDE..AVAYVKSLQKQIE........EL61
AhbHLH082.1  RRGQATDPHSIAER...LRRERIA......ERMKALQELVPN.........ANK.................TDKASMLDE..IIDYVKFLQLQVK........VL60
AhbHLH082.2  RRGQATDPHSIAER...LRRERIA......ERMKALQELVPN.........ANK.................TDKASMLDE..IIDYVKFLQLQVK........VL60
AhbHLH084.1  SRGSATDPQSIYAR...RRRERIN......ERLRILQNLVPN.........GTK.................VDISTMLEE..AVQYVKFLQLQIK........VI60
AhbHLH084.2  SSGAATDPQSLYAR...KRRERIN......ERLRILQTLVPN.........GTK.................VDISTMLEE..AVHYVKFLQLQIK........LL60
AhbHLH084.3  SSGAATDPQSLYAR...KRRERIN......ERLRILQTLVPN.........GTK.................VDISTMLEE..AVHYVKFLQLQIK........LL60
AhbHLH084.4  SRGSATDPQSIYAR...RRRERIN......ERLRILQNLVPN.........GTK.................VDISTMLEE..AVQYVKFLQLQIK........LL60
AhbHLH086.1  KLGASKDPQSVAAK...NRRERIS......ERLKILQELVPN.........GSK.................VDLVTMLEK..AISYVKFLQLQVK........VL60
AhbHLH086.2  KSGASKDPQSVAAK...NRRERIS......ERLKILQELVPN.........GSK.................VDLVTMLEK..AISYVKFLQLQVK........VL60
AhbHLH086.3  KSEASKDPQSVAAK...NRKEGIS......ERLNILQELVPN.........GSK.................VDLVIMLEK..AISYVKFLQLQVK........VL60
AhbHLH087.1  NVKISSDPQTVAAR...QRREKIS......ERIRVLQKIVPG.........GSK.................MDTASMLDE..AANYLKFLRSQVK........AL60
AhbHLH087.2  NVRVSSDPQTVAAR...QRREKIS......QKIRVLQKIVPG.........GTK.................MDTASMLDE..AANYLKFLRSQVK........AL60
AhbHLH087.3  NVKISSDPQTVAAR...QRREKIS......ERIRVLQKIVPG.........GSK.................MDTASMLDE..AANYLKFLRSQVK........AL60
AhbHLH087.4  ....SSDPQTVAAR...QRREKIS......QKIRVLQKIVPG.........GTK.................MDTASMLDE..AANYLKFLRSQVK........AL56
AhbHLH090.1  KEGYHA.KNLATER...RRRNKIK......NGLFTLRSLVP..........KITK................MDRASILAD..AIDYIKELHGQVE........DL59
AhbHLH090.2  KEGYHA.KNLATER...RRRNKIK......NGLFTLRSLVP..........KITK................MDRASILAD..AIDYIKELHGQVE........DL59
AhbHLH091.1  NNNNKQFSSTNTER...QRRVDLG......GKFDALKELIPSS.........TKN.................DRASVVGD..AIDYIKELLRTVN........EL60
AhbHLH091.2  ....KGTKHFATEK...QRREQLN......GKYKILRSLIPSP.........TKM.................DRASVVGD..AIEYIRELLRTVN........EL56
AhbHLH091.3  GRGRRGQGTMPIDK...QRREQLN......DKYQILRSLIPSP.........TKV.................DRASVVGD..AIDYIRELIRTVN........EL60
AhbHLH091.4  NNNIKQFSSTNTER...QRRVDLG......GKFDALKELIPSS.........TKN.................DRASVVGD..AIDYIKELLRTVN........EL60
AhbHLH091.5  ....KGTKHFATEK...QRREQLN......GKYKILRSLIPSP.........TKM.................DRASVVGD..AIEYIRELLRTVN........EL56
AhbHLH091.6  GRGRRGQGTMPIDK...QRREQLN......DKYQILRSLIPSP.........TKV.................DRASVVGD..AIDYIRELIRTVN........EL60
AhbHLH091.7  QLNFATEKHFATEK...QRREQLN......GKYKILRSLIPSP.........TK..................ALLQALGDRKGINRFGDFSAPLD........EA61
AhbHLH093.1  .KEREAYMSSRG.....RKKAAMQ......RMLQQLRTATN..........SSA.................MNKASIIVD..ATKYMEELKQKVE........GI56
AhbHLH093.2  ......MASSRE.....RRREALQ......QKLNQLRDVTN..........SSG.................VNKASIIVD..ASRYIEELKQKVE........GL51
AhbHLH093.3  ......MVSREQ.....KRAALLH......EKLQLLRSVTN..........SHA.................VNKSSIIID..ASKYIEELKEKVE........RL51
AhbHLH093.4  ......MVSREQ.....KRAALLH......EKLQLLRSITN..........SHA.................LNKTSIIID..ASKYIRDLKQKVE........RL51
AhbHLH093.5  KKEREAYMSSRG.....RKKAAMQ......RMLQQLRTATN..........SSA.................MNKASIIVD..ATKYIEELKQKVE........GI57
AhbHLH093.6  ......MASSRE.....RRREALQ......QKLNQLRDVTN..........SSG.................VNKASIIVD..ASRYIEELKQKVE........GL51
AhbHLH093.7  ......MVSREQ.....KRAALLH......EKLQLLRSITN..........SHA.................VNKTSIIID..ASKYIEELKEKVE........RL51
AhbHLH093.8  ......MVSREQ.....KRAALLH......EKLQLLRSITN..........SHA.................LNKTSIIID..ASKYIRDLKQKVE........RL51
AhbHLH095.1  GDSECGGSSMPAER...NRRLKMN......RLFTQLQATIPR.........PLSK................ATKEVVITE..TIRYIKELERKKN........NL61
AhbHLH095.2  NDGNDHDLHIWTER...ERRKKMR......DMFANLHAMLPQ.........LPPK................ADKSSIVDE..AVRHIKTLQQTVE........NL61
```

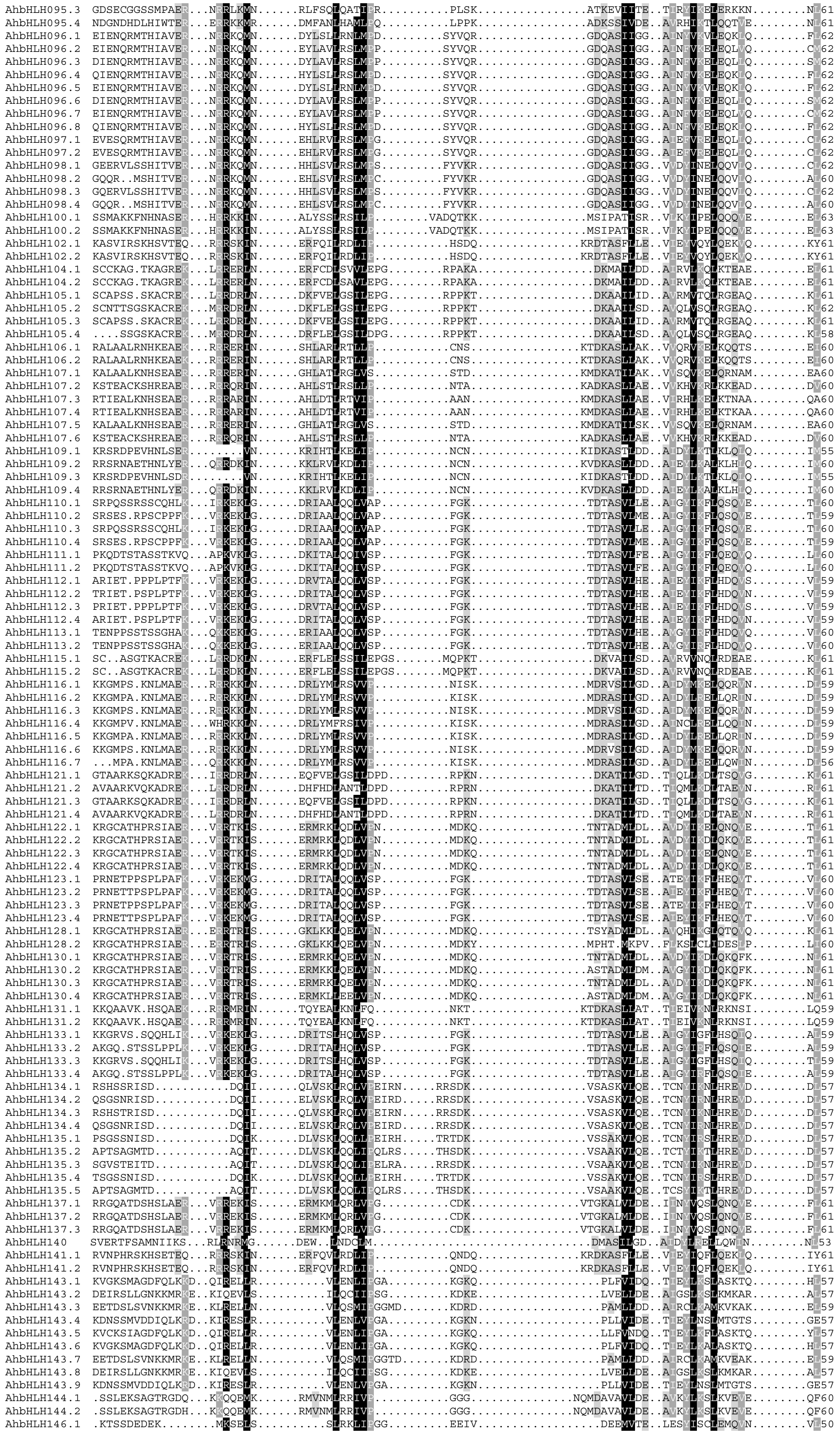
AhbHLH095.3
AhbHLH095.4
AhbHLH096.1
AhbHLH096.2
AhbHLH096.3
AhbHLH096.4
AhbHLH096.5
AhbHLH096.6
AhbHLH096.7
AhbHLH096.8
AhbHLH097.1
AhbHLH097.2
AhbHLH098.1
AhbHLH098.2
AhbHLH098.3
AhbHLH098.4
AhbHLH100.1
AhbHLH100.2
AhbHLH102.1
AhbHLH102.2
AhbHLH104.1
AhbHLH104.2
AhbHLH105.1
AhbHLH105.2
AhbHLH105.3
AhbHLH105.4
AhbHLH106.1
AhbHLH106.2
AhbHLH107.1
AhbHLH107.2
AhbHLH107.3
AhbHLH107.4
AhbHLH107.5
AhbHLH107.6
AhbHLH109.1
AhbHLH109.2
AhbHLH109.3
AhbHLH109.4
AhbHLH110.1
AhbHLH110.2
AhbHLH110.3
AhbHLH110.4
AhbHLH111.1
AhbHLH111.2
AhbHLH112.1
AhbHLH112.2
AhbHLH112.3
AhbHLH112.4
AhbHLH113.1
AhbHLH113.2
AhbHLH115.1
AhbHLH115.2
AhbHLH116.1
AhbHLH116.2
AhbHLH116.3
AhbHLH116.4
AhbHLH116.5
AhbHLH116.6
AhbHLH116.7
AhbHLH121.1
AhbHLH121.2
AhbHLH121.3
AhbHLH121.4
AhbHLH122.1
AhbHLH122.2
AhbHLH122.3
AhbHLH122.4
AhbHLH123.1
AhbHLH123.2
AhbHLH123.3
AhbHLH123.4
AhbHLH128.1
AhbHLH128.2
AhbHLH130.1
AhbHLH130.2
AhbHLH130.3
AhbHLH130.4
AhbHLH131.1
AhbHLH131.2
AhbHLH133.1
AhbHLH133.2
AhbHLH133.3
AhbHLH133.4
AhbHLH134.1
AhbHLH134.2
AhbHLH134.3
AhbHLH134.4
AhbHLH135.1
AhbHLH135.2
AhbHLH135.3
AhbHLH135.4
AhbHLH135.5
AhbHLH137.1
AhbHLH137.2
AhbHLH137.3
AhbHLH140
AhbHLH141.1
AhbHLH141.2
AhbHLH143.1
AhbHLH143.2
AhbHLH143.3
AhbHLH143.4
AhbHLH143.5
AhbHLH143.6
AhbHLH143.7
AhbHLH143.8
AhbHLH143.9
AhbHLH144.1
AhbHLH144.2
AhbHLH146.1

```
AhbHLH146.2  .KTSSDEDEK........MKSQLS..........SLRKLIPGG.........EEIV.................DEEMVTE..LESYISCLEMQVN........VL50
AhbHLH147.1  .SVLRLKGKKNLP....AVQRKLR..........FLARLVPGC.........RKEP.................VPVILEE..AIDYIPALEMQVR........AM54
AhbHLH147.2  .NVVRLK.VKTVP....AVQKKVR..........LLGRLVPGC.........RKEP.................LPVILEE..AIDYIPALEMQVR........AM53
AhbHLH147.3  .SVLRLKGKKNLP....AVQRKLR..........FLARLVPGC.........RKEP.................VPVILEE..AIDYIPALEMQVR........AM54
AhbHLH147.4  .NVVRLK.VKTVP....AVQKKVR..........LLGRLVPGC.........RKEP.................LPVILEE..AIDYIPALEMQVR........AM53
AhbHLH149.1  ..KKSPESRRRLP....VVQKKAR..........VLGRLVPGC.........RKLT.................FPNLLEE..ATDYISALEMQVR........AM53
AhbHLH149.2  .SMRNGTEKKKLP....AVERKTR..........VLSRLVPGC.........RKLS.................FPNLLEE..ATDYISALEMQVR........AM54
AhbHLH149.3  ..KKSPETRRRLP....VVQKKAR..........VLGRLVPGC.........RKLT.................FPNLLEE..ATDYISALEMQVR........AM53
AhbHLH149.4  .SMRNGTEKKKLP....AVQRKAR..........VLSRLVPGC.........RKLS.................FPNLLEE..ATDYISALEMQVR........AM54
AhbHLH151    ......RSRGRGTK....SNNGIQ......SRVRTLKNLIPN..........SDNN...............MGLDGLFRE..TANYILSLQNRVR........VM54
AhbHLH153.1  SKRQKANDLSITTK...ERKEKLS......ERIVALQQLVSP..........YGK................TDTSSVLEE..AMQYIRFLHKQVK........LL60
AhbHLH153.2  SKRQKANDLSITAK...ERKEKLS......ERIVALQQLVSP..........YGK................TDTSSVLEE..AMQYIGFLHRQVK........LL60
AhbHLH155.1  KNSKKRARPGESCRPRPRDRQLIQ......DRIKELRELVP...........NGAK...............CSIDSLLEC..TIKHMLFLQSVTK........HA63
AhbHLH155.2  STYKKRARPG.TQKSRPRDRQLIM......DRMKELRELVP...........DGGK...............CSIDNLLER..TIKHMLYLRKITS........QA62
AhbHLH155.3  KNSKKRARPGESCRPRPRDRQLIQ......DRIKELRELVP...........NGAK...............CSIDSLLEC..TIKHMLFLQSVTK........HA63
AhbHLH155.4  STYKKRARPG.TQKSRPRDRQLIM......DRMKELRELVP...........DGGK...............CSIDNLLER..TIKHMLYLRKITS........QA62
AhbHLH156.1  KSNRKRLKPGENPRPRPKDRQMIQ......DRVKELREIVP...........NGAK...............CSIDSLLER..TIKHMLFLQSVTK........HA63
AhbHLH156.2  KSNRKRLKPGENPRPRPKDRQMIQ......DRVKELREIVP...........NGAK...............CSIDALLER..TIKHMLFLQSVTK........HA63
AhbHLH156.3  KSNRKRLKPGENPRPRPKDRQMIQ......DRVKELREIVP...........NGAK...............CSIDSLLER..TIKHMLFLQSVTK........HA63
AhbHLH156.4  KSNRKRLKPGENPRPRPKDRQMIQ......DRVKELREIVP...........NGAK...............CSIDALLER..TIKHMLFLQSVTK........HA63
AhbHLH157.1  KPSKKKDKTGECSRPRPKDRQRIQ......DCIQQLRTIIPGK.........HVKE...............CSIDNLLEC..SIRYMSFLSSTAH........YA65
AhbHLH157.2  KPSKKKDKAGVGSRPRPKDRQRIQ......DCIRQLRTIIPGK.........NVKE...............CSIDNLLEC..SIRYMSFLSSTAH........YA65
AhbHLH159.1  KSAKKEMSASCIAKR..LVEKRTR..........KLKNLLPGG.........ESID.................EGCLVEE..TLDYIESLRAQIE........VM57
AhbHLH159.2  KSAKKEMSASCIAKR..LAEKRTR..........KLKNLLPGG.........ESID.................EGCLVEE..TLDYIESLRAQIE........VM57
AhbHLH161.1  SSGGSEFTE...........NEIN......VLASRLQALLPQPSQQ....TRNSRQ...............VSVLEILKE..TCSHIKRLQKDVE........DL59
AhbHLH161.2  SFLPKPARQKASDRVSQQRRQRIA......DNLKALHDLLPN..........QAEG................SQAYILDD..IIDHVKYLQLQLK........EL63
AhbHLH161.3  SSGRSEFTE...........NEIN......DLASRLQALLPQPNQQ....TRNSR................VSVLEILKE..TCSHIKRLQKDVE........DL58
AhbHLH161.4  SFLPKPARQKASDRVSQQRRQRIA......DNLKALHDLLPN..........QAEG................SQAYILDD..IIDHVKYLQLQLK........EL63
AhbHLH161.5  HFIWQYGSRYLFVRCDNEPAPWIA......DILKALHDLLPN..........QAEG................SQAYILDD..IIDHVKYLQLQLK........EL63
AhbHLH162.1  STNKKKIERRVIEK...NRRNHMK......MLYSKLNSLLP.........RPKEPL...............PL.PDQVDK..VINYIKSLEEKVK........MA61
AhbHLH162.10 SSTTNKIERRLIEK...NRRNHMK......MLYSKLNSLLPN.......YNPKEAL...............PL.PDQVDE..AINYIKSLEEKVK........TA63
AhbHLH162.11 STNDKKIERRLIEK...KRRKHMR......MLYSKLNALLPIDH...NSTSPKEVL...............PL.PDQVDK..AINYIKNLKKKVK........MA67
AhbHLH162.12 SNEPSKLDRKTIER...NRRIHMK......SLCFKLTSLIPPNHRFTQYSKSKDTL...............TQ.QDQLDL..AARYIKHMRERIE........EL70
AhbHLH162.13 PSTTTKVERKIIER...NRRNKMK......DLYSKLNSLLPS.......YSPTEVL...............PL.PDQIDE..AVKYIKSLEKKVK........MA63
AhbHLH162.14 ASSSTKMERKIIER...NRREQMK......NLCFKLNSLLPN.......FNPKQTL...............PPRVDQIDE..AIIYIKTLESRVE........IA64
AhbHLH162.15 STCSTRTERKFIER...ERRNQMK......ALYSNLNSLLPP.......QTSRERI...............SSLPDQLEE..ATNYIKKLQVKLE........KM64
AhbHLH162.2  SSTTNKIERRLIEK...NRRNHMK......MLYSKLNSLLPN.......YNPKEAL...............PL.PDQVDE..AINYIKNLEEKVK........TA63
AhbHLH162.3  SSSSKKDDRRLIES...HRRILMK......NLYSKLNSLQPN.......SNTKKAL...............P...DQLDE..AINYIKSLEEKVK........MA61
AhbHLH162.4  STNKEKIERRFIER...NRRKHMK......MLYSTLNSLLPNDP...NPTRPKEAL...............LL.LDQVDK..ALNYIKSLEEKVK........MS67
AhbHLH162.5  SNEPSKLDRKTIER...NRRIHMK......SLCFKLTSLIPPNHRFTQYSKSKETL...............TQ.QDQLDL..AARYIKHMRERIE........EL70
AhbHLH162.6  ASSSTKMERKIIER...NRREQMK......NLCFTLNSLLPN.......FNPKQTL...............PPRVDQIDE..AIIYIKTLESRVE........IA64
AhbHLH162.7  PSTTTKVERKIIER...NRRNKMK......DLYSKLNSLLPS.......YSPTEVL...............PL.PDQIDE..AVKYIKSLEKKVK........MA63
AhbHLH162.8  STCSTRTERKFIER...ERRSQMK......ALYSNLNSLLPP.......QTSREGI...............SSLPDQLEE..ATNYIKKLQVKLE........KM64
AhbHLH162.9  STNKKKIERRVIEK...NRRNHMK......MLYSKLNSLLP.........RPKEPL...............PL.PDQVDK..AINYIKSLEEKVK........MA61
```

Figure S1. Sequence alignment of AhbHLHs with ClustalW. Identical residues are highlighted in black and homologous residues in gray.
